# Temporal analysis of type 1 interferon activation in tumor cells following external beam radiotherapy or targeted radionuclide therapy

**DOI:** 10.7150/thno.54881

**Published:** 2021-04-15

**Authors:** Justin C. Jagodinsky, Won Jong Jin, Amber M. Bates, Reinier Hernandez, Joseph J. Grudzinski, Ian R. Marsh, Ishan Chakravarty, Ian S. Arthur, Luke M. Zangl, Ryan J. Brown, Erin J. Nystuen, Sarah E. Emma, Caroline Kerr, Peter M. Carlson, Raghava N. Sriramaneni, Jonathan W. Engle, Eduardo Aluicio-Sarduy, Todd E. Barnhart, Trang Le, KyungMann Kim, Bryan P. Bednarz, Jamey P. Weichert, Ravi B. Patel, Zachary S. Morris

**Affiliations:** 1 Department of Human Oncology, University of Wisconsin School of Medicine and Public Health, Madison, WI.; 2 Department of Radiology, University of Wisconsin School of Medicine and Public Health, Madison, WI.; 3 Department of Medical Physics, University of Wisconsin School of Medicine and Public Health, Madison, WI.; 4 Department of Biostatistics and Medical Informatics, University of Wisconsin School of Medicine and Public Health, Madison, WI.; 5 Department of Radiation Oncology, University of Pittsburgh School Hillman Cancer Center, Pittsburgh, PA.

**Keywords:** type 1 interferon, immune susceptibility, targeted radionuclide therapy, external beam radiotherapy, checkpoint blockade

## Abstract

**Rationale:** Clinical interest in combining targeted radionuclide therapies (TRT) with immunotherapies is growing. External beam radiation therapy (EBRT) activates a type 1 interferon (IFN1) response mediated via stimulator of interferon genes (STING), and this is critical to its therapeutic interaction with immune checkpoint blockade. However, little is known about the time course of IFN1 activation after EBRT or whether this may be induced by decay of a TRT source.

**Methods:** We examined the IFN1 response and expression of immune susceptibility markers in B78 and B16 melanomas and MOC2 head and neck cancer murine models using qPCR and western blot. For TRT, we used ^90^Y chelated to NM600, an alkylphosphocholine analog that exhibits selective uptake and retention in tumor cells including B78 and MOC2.

**Results:** We observed significant IFN1 activation in all cell lines, with peak activation in B78, B16, and MOC2 cell lines occurring 7, 7, and 1 days, respectively, following RT for all doses. This effect was STING-dependent. Select IFN response genes remained upregulated at 14 days following RT. IFN1 activation following STING agonist treatment *in vitro* was identical to RT suggesting time course differences between cell lines were mediated by STING pathway kinetics and not DNA damage susceptibility. *In vivo* delivery of EBRT and TRT to B78 and MOC2 tumors resulted in a comparable time course and magnitude of IFN1 activation. In the MOC2 model, the combination of ^90^Y-NM600 and dual checkpoint blockade therapy reduced tumor growth and prolonged survival compared to single agent therapy and cumulative dose equivalent combination EBRT and dual checkpoint blockade therapy.

**Conclusions:** We report the time course of the STING-dependent IFN1 response following radiation in multiple murine tumor models. We show the potential of TRT to stimulate IFN1 activation that is comparable to that observed with EBRT and this may be critical to the therapeutic integration of TRT with immunotherapies.

## Introduction

Radiation can activate a type 1 interferon (IFN1) response in tumor cells, and in preclinical studies this has been shown to be critical to the role of radiation in activating an anti-tumor immune response in combination with immune checkpoint inhibition (ICI, eg. anti-CTLA-4, anti-PD-1/PD-L1) 1-10. IFN1 production, mediated through activation of cyclic GMP-AMP (cGAMP) synthase (cGAS) and its downstream adaptor stimulator of interferon genes (STING), plays a key role in recruitment and activation of dendritic cells as well as CD8 T cell cross priming 11. Resulting at least in part from these mechanisms, preclinical and clinical studies demonstrate that external beam radiation therapy (EBRT) targeting a single tumor can convert the targeted tumor into a site for enhanced antigen presentation, stimulating T cell recognition of a greater diversity of tumor antigens 4, 10. Consequently, EBRT can improve the response of immunologically hot or cold tumors to immune checkpoint inhibition with anti-PD-1 or anti-CTLA-4 in murine models 7, 12, 13. However, in some tumor types such as prostate cancer EBRT has been reported to recruit immune suppressive lineages, at least transiently to the tumor 14. Despite this potentially immune suppressive effect, EBRT has been reported to improve the response to immune checkpoint blockade 15.

Prior studies further indicate that tumor cells surviving radiation may become more susceptible to immune mediated elimination in part by altering the expression of several surface proteins identified as immune susceptibility markers 16-18. These markers when upregulated enable more effective immune mediated elimination of tumor cells through a variety of mechanisms. Death receptors such as Fas and DR5 as well as antigen presentation machinery, such as MHC-1 have been implicated as markers of immune susceptibility following radiation 17, 19-21. Additionally, expression of the immune checkpoint ligand PD-L1 has been shown to be influenced by radiation 1, 22. The time course, dose and dose-rate responsiveness, and potentially the shared underlying mechanisms of radiation mediated effects on IFN1 activation and expression of immune susceptibility markers remain to be clarified.

Targeted radionuclide therapy (TRT) is a class of cancer therapeutics that use a vector or biologic mechanism to selectively deliver a radionuclide to the tumor microenvironment (TME), where, upon its decay, that radionuclide will deliver radiation to the tumor. This therapeutic approach may be particularly beneficial in the metastatic setting as TRT can deliver radiation to all sites of disease including micro-metastases. For many patients with metastatic disease, this would not be feasible using EBRT approaches without also inducing lymphopenia. TRTs are in development for nearly all cancers 23-28. Few preclinical studies have investigated the efficacy of TRT agents in combination with ICI 29-31. However, it is largely unknown what effect TRT may have on response to immune checkpoint inhibitors or how the continuous, low-rate, dose deposition of TRT will affect the activation of IFN1 response and expression of immune susceptibility markers in tumor cells, as compared to EBRT. Here we report on the time course and magnitude of IFN1 activation and changes in the expression of immune susceptibility markers following EBRT and TRT *in vitro* and *in vivo*. We then test the functional consequences of these effects of TRT by evaluating the capacity of a novel TRT agent, ^90^Y-NM600, to augment response to immune checkpoint blockade in an immunologically cold syngeneic murine model of head and neck cancer 32, 33.

## Materials and methods

### Cell lines

The murine head and neck cancer MOC2 cell line was generously provided by Dr. Ravindra Uppaluri (Dana-Farber Cancer Institute). Wild-type (WT) murine B16 melanoma and *Tmem173* -/- CRISPR deletion B16 (STING KO) melanoma cell lines were generously provided by Dr. Samuel Bakhoum (Memorial Sloan Kettering Cancer Center). The murine melanoma B78-D14 (B78) cell line, derived from B16 melanoma as previously described, was obtained from Ralph Reisfeld (Scripps Research Institute) in 2002 34. B78 and MOC2 cells were grown in RPMI-1640 and were supplemented with 10% FBS, 100 U/mL penicillin, and 100 µg/mL streptomycin. Wild-type murine melanoma B16 and STING knock out B16 cell lines were grown in DMEM and were supplemented with 10% FBS, 100 U/mL penicillin, and 100 ug/mL streptomycin. Cell line authentication was performed per ATCC guidelines using morphology, growth curves, and Mycoplasma testing within 6 months of use.

### Cell culture

EBRT was delivered when cells were approximately 60-70% confluent. Following radiation, cell media was exchanged with fresh pre-warmed media every day in both radiation and control plates until cell harvest to remove dead nonadherent cells and cellular debris. Only adherent cells were collected and analyzed for *in vitro* studies. Cells were incubated in a humidified incubator at 37 °C with 5% CO_2_.

### Murine tumor models

Mice were housed and treated under a protocol (protocol number M005670) approved by the Institutional Animal Care and Use Committee (IACUC) at the University of Wisconsin - Madison. Female C57BL/6 mice were purchased at age 6 to 8 weeks from Taconic. B78 and MOC2 tumors were engrafted by subcutaneous flank injection of 2 × 10^6^ tumor cells. Tumor size was determined using calipers and volume approximated as (width^2^ × length)/2. Mice were randomized immediately before treatment using a randomly generated treatment list (GraphPad Prism 8). Treatment began when tumors were well-established (100- 150 mm^3^), which occurred approximately 4 weeks after tumor implantation for B78 and 2 weeks for MOC2. The day of radiation was defined as “day 1” of treatment. In the case of the metastatic model, the secondary tumor was injected 1 week after the primary. Anti-CTLA-4 (IgG2c, clone 9D9, provided by Bristol Myers Squibb) and anti-PD-L1 (IgG2b, clone 10F.9G2, Bio-X-cell) was administered by 200 µg intraperitoneal injection on days 4, 7, and 10. Mice were euthanized when tumor size exceeded 15 mm in longest dimension or whenever recommended by an independent animal health monitor for morbidity or moribund behavior.

### Imaging and biodistribution

Mice bearing B78 and MOC2 (n=3) flank tumors (100-150 mm^3^) were injected via tail vein with 9.25 MBq of ^86^Y-NM600 and imaged with an Inveon microPET/microCT scanner (Siemens Medical Solutions, Knoxville, TN) at 3, 24, 48, and 72 hours post injection of the radiotracer. For each scan, mice were anesthetized with isoflurane and placed in the prone position on the scanner bed. Sequential CT (80 kVp; 1000 mAs; 220 angles) and static PET scans (80 million coincidence events; time window: 3.432 ns; energy window: 350-650 keV) were collected. A three-dimensional ordered subset expectation maximization algorithm was used to reconstruct the PET images. These were then fused with corresponding CT images for attenuation correction and anatomical referencing. Tumor and organs of interest were contoured for region-of-interest analysis of the PET images to determine the magnitude and kinetics of ^86^Y-NM600 uptake, which is reported as percent injected activity per gram of tissue (%IA/g; mean ± SD). To verify the accuracy of the image-derived quantification, ex-vivo biodistibution analysis was performed. For this mice were euthanized after the last PET/CT image was acquired. Tumors and normal tissue specimens were collected, wet-weighed, counted in an automated γ-counter (Wizard 2, Perking Elmer, MA), and the %IA/g (mean ± SD) corresponding to each tissue was calculated.

### *In vivo* dosimetry estimation

Murine-specific 3D cumulative dose distributions were estimated using the RAPID Monte Carlo‐based dosimetry assessment platform 35-38. PET/CT volumes were processed in the Monte Carlo framework (Geant4 version 9.6) as previously described 35-38. Briefly, at each time point by correcting for differences in physical decay rates ^86^Y‐NM600 activity concentration was converted to ^90^Y‐NM600. CT images were used to define the geometry and PET images were used to define the source distribution. Using a combination of trapezoidal and analytical integration, the dose rate in each volume of interest was integrated over time to calculate cumulative dose. Tumor and organs of interest were contoured on the CT images and used to quantify the spatial distribution of the absorbed dose imparted by ^90^Y‐NM600.

### Radiation

Delivery of EBRT *in vitro* was performed using a RS225 Cell Irradiator (Xstrahl). Delivery of EBRT *in vivo* was performed using an X-ray biological cabinet irradiator X-RAD 320 (Precision X-Ray, Inc). EBRT was prescribed to 2.5 Gy, 12 Gy, or 3 fractions of 8 Gy. For the 8 Gy × 3 fraction regimen, cells or tumors were radiated once per day for 3 consecutive days. Day 1 following radiation was defined as 24 hours following the last fraction of radiation. The dose rate for EBRT delivery in all experiments was approximately 2 Gy/min. Dosimetric calibration and monthly quality assurance checks were performed on these irradiators by University of Wisconsin Medical Physics Staff.

For TRT, we used ^90^Y conjugated to NM600, an alkylphosphocholine analog that exhibits selective uptake and retention in tumor cells of nearly any type, including B78 24, 37, 39. For time course studies ^90^Y-NM600 was injected with an activity of either 20 µCi or 100 µCi for MOC2, and 50 µCi or 250 µCi for B78, which corresponds to ~ 2.5 Gy or 12 Gy cumulative tumor absorbed dose. For survival analysis in combination with anti-CTLA-4, ^90^Y-NM600 was injected with an activity of 100 µCi.

### Gene expression analysis

Cells radiated *in vitro* were washed with cold PBS, TRIzol^TM^ reagent (ThermoFisher Scientific Cat # 15596026) was added to the plate, and the cells were collected via scraping over ice. For analysis of tumor tissue, tumors were harvested and samples were homogenized in TRIzol using a Bead Mill Homogenizer (Bead Ruptor Elite, Omni International Cat # 19-040E). For *in vitro* and *in vivo* samples, total RNA was extracted using RNeasy Mini Kit (QIAGEN, Germany, Cat # 74106) according to the manufacturer's instructions. Extracted RNA was subjected to complementary cDNA synthesis using QuantiTect Reverse Transcription Kit (QIAGEN, Germany, Cat # 205314) according to the manufacturer's instructions. Quantitative polymerase chain reaction (qRT-PCR) was performed using PowerUp SYBR Green qPCR Master Mix. The reaction (5 µL total volume) was prepared using Echo 550 (Labcyte) and TEMPEST (Formulatrix) liquid handling systems. Thermal cycling conditions (Quantstudio 6, Applied Biosystems) included the UDG activation at 50 °C for 2 min, followed by Dual-Lock™ DNA polymerase activation stage at 95 °C for 2 min followed by 40 cycles of each PCR step (denaturation) 95 °C for 15s and (annealing/extension) 60 °C for 1 min. A melt curve analysis was also done to ensure the specificity of the corresponding qRT-PCR reactions. For data analysis, the Ct values were exported to an Excel file and fold change normalized to untreated control samples was calculated using the ∆∆Ct method. *Hprt*, *Pgk1*, and *Tbp* were used as endogenous controls. A complete list of primer sequences is included as a supplement (Table S1).

### Clonogenic assay

*In vitro* clonogenic assay of B78 and MOC2 cells was performed as previously described 21. Briefly, exponentially growing cells in monolayer culture were irradiated with either 0 Gy, 3 Gy, 6 Gy or 9 Gy. After irradiation, cells were harvested and replated for clonogenic survival analysis. Surviving colonies were stained with crystal violet to aid colony counting. Colonies containing >50 cells were scored to determine plating efficiency and the fractions of the cells surviving after each radiation dose. The log surviving fraction of control and irradiated colonies were calculated and plotted.

### STING agonist treatment

B78 and MOC2 cells were transfected *in vitro* with 2 µg G3-YSD 40 (Invivogen) using Lipofectamine 3000 (Thermofisher) per manufacturer's instructions. Control samples were transfected with Lipofectamine 3000 vehicle only. Additionally, B78 and MOC2 cells were treated with 200 nM of the small molecule STING agonist diABZI 41. Cells were harvested either 24 hours or 7 days following transfection for analysis of IFN1 expression.

### Western blot

Protein isolation, quantitation, and immunoblotting were performed as previously described 15. Briefly, proteins from B78 and MOC2 cell lines were extracted in RIPA buffer (Thermo Scientific, Cat # 89900) containing protease inhibitor cocktail (Thermo Scientific, Cat # 78444). Protein concentrations were determined via Pierce™ BCA Protein Assay (Thermo Scientific, Cat # 23227). A total of 35 µg of protein of each sample was loaded in 10% SDS-PAGE and run at 150 V constant voltage. Transfers of protein on an activated PVDF membrane (Millipore, Cat # IPVH07850) were performed for 2 hours at 4 °C at 50 mA. Ponceau staining was used to confirm transfer and was washed out prior to antibody probing. PVDF membranes were probed with primary antibodies (mouse anti-phospho-gH2A.X, Cell Signaling Technology, Cat # 9718S, 1:1,000; mouse anti-vinculin, Cell Signaling Technology, Cat # 13901S, 1:1,000) at 4 °C overnight. After washing three times, blots were then incubated with HRP goat anti-rabbit IgG secondary antibody (Biorad - Cat # 1706515; 1:10,000) at room temperature for 1 hour. Chemiluminescence was used to visualize protein bands (Thermo Scientific, Cat # 34076). Pictures were acquired using the Odyssey® Fc imaging system (Li-cor). Precision Plus Protein Dual Colour standard was used to estimate molecular weight (BioRad - Cat # 1610374).

### *In vitro* dosimetry

Thermoluminescent dosimeters (TLDs) were placed in the bottom of 60 mm culture plates and a serial dilution of known ^90^Y-NM600 activity concentration was added to the plates. TLDs were harvested after 1 half-life (64.1 hrs) and analyzed by the University of Wisconsin-Madison Radiation Calibration Laboratory (Calibration Cert # 1664.01). A standard curve was generated and used to calculate the required amount of ^90^Y-NM600 activity for *in vitro* studies.

### *In vitro* co-culture

B16 WT, B16 STING KO, or MOC2 cells were plated in 12 well plates (50,000 cells per well) and irradiated with either 12 Gy of EBRT or 12 Gy cumulative absorbed dose of ^90^Y-NM600 (140 uCi diluted in 2 ml complete growth media). Three days following irradiation spleens from naïve C57BL/6 mice were harvested, dissociated into a single cell suspension, and added to the tumor cells (2×10^6^ cells per well). One day following splenocyte addition, cells were harvested and analyzed via flow cytometry.

### Flow cytometry

Harvested cells for analysis were treated with GolgiStop™ protein transport inhibitor (BD Bioscience) for 5 hours before antibody staining. Total cells were harvested and treated CD16/32 antibody (BioLegend) to prevent tumor cell non-specific binding. Flow cytometry was performed as previously described 42, using fluorescent beads (UltraComp Beads eBeads, 176 Invitrogen, #01-2222-42) to determine compensation and fluorescence minus one (FMO) methodology to determine gating. Live cell staining was performed using Ghost Red Dye 780 (Tonbo Biosciences) according to manufacturer's instruction. Antibodies used for flow cytometry include: anti-CD45-PE-Cy7 (BioLegend), anti-CD3-FITC (BioLegend), CD4-BV510 (BioLegend), CD8-PerCP-Cy5.5 (BioLegend), and anti-IFNγ-APC (BioLegend). After live-dead staining, a single cell suspension was labeled with the surface antibodies at 4 °C for 30 min and washed three times using flow buffer (2% FBS + 2 mM EDTA in PBS). For intracellular staining, the cells were fixed and stained for internal IFNγ with permeabilization solution according to manufacturer's instructions (BD Cytofix/CytopermTM). Flow cytometry was performed using an Attune NxT Flow Cytometer (ThermoFisher). Data was analyzed using FlowJo Software.

### Statistical analysis

Prism 8 (GraphPad Software) and R version 4.0.2 (The R Foundation) were used for all statistical analyses. Student's t-test was used for two-group comparisons. Two-way ANOVA with Tukey's honestly significant difference (HSD) test to adjust for multiple comparisons was used to assess statistical significance of observed mean differences in gene expression. For comparisons between two groups a Student's t-test was performed. For tumor growth analysis, a linear mixed model after log transformation of tumor volume was fitted in which TRT, CTLA-4 and day and two-way and three-way interactions as fixed effects. A complete case analysis was used, which discards only the missing measurements, to handle the missing data. Kaplan-Meier method was used to estimate the survival distribution for the overall survival. A Cox regression model was fitted with TRT and CTLA-4 as fixed effects. Then, pairwise comparison of the overall survival was made using a log-rank test with Benjamini-Hochberg adjustment of p-values between levels of factors. All data presented is reported as mean ± SEM unless otherwise noted. For all graphs, *, P < 0.05; **, P < 0.01; ***, P < 0.001; and ****, P < 0.0001.

## Results

### STING-dependent activation of IFN1 following *in vitro* radiation is dose, time, and cell-line dependent

**Figure 1:**
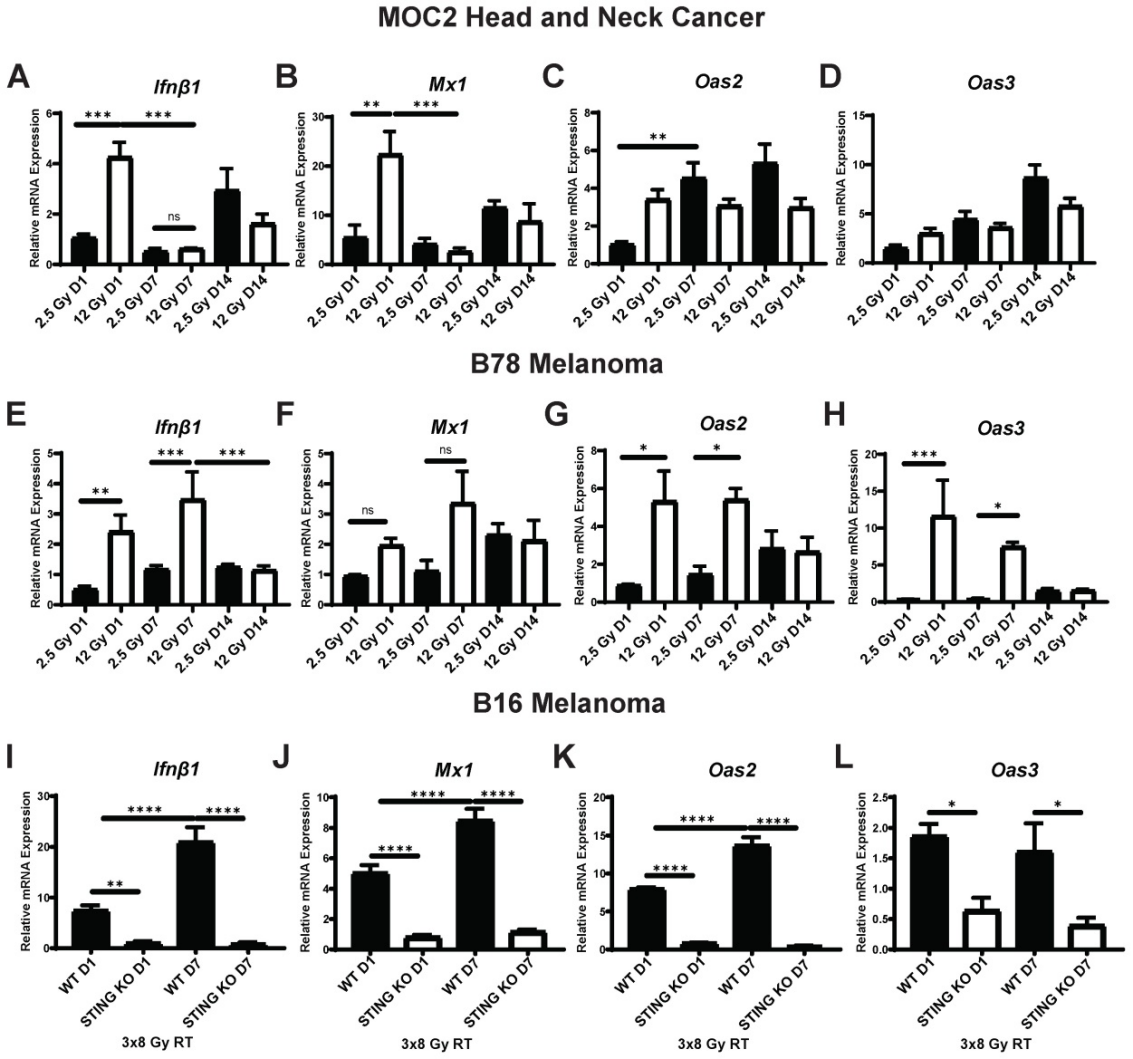
Time course of STING-dependent IFN1 activation displays unique kinetics in murine models of head and neck cancer and melanoma following EBRT *in vitro*. (A-H) Cells were radiated with either 2.5 Gy or 12 Gy of EBRT and harvested 1, 7, or 14 days following radiation. qPCR was used to quantify gene expression and is reported as fold change normalized to untreated controls. (I-L) WT and STING KO B16 melanoma cells were radiated with 3 daily fractions of 8 Gy and harvested either 1 or 7 days following the last fraction of radiation. N=5 per treatment group per timepoint. Two-way ANOVA with Tukey's HSD post hoc test was used to compare fold change in expression between groups. * indicates p-value < 0.05, ** indicates p-value < 0.01, *** indicates p-value < 0.001, and **** indicates p-value < 0.0001.

To investigate the time course of IFN1 activation following radiation we measured the effect of low and moderate dose EBRT, 2.5 Gy and 12 Gy respectively, on expression of *Ifnβ1* as well as interferon stimulated genes *Mx1*, *Oas2*, and *Oas3 in vitro*. We initially omitted investigation of higher doses of EBRT as it has been reported that higher dose per fraction above 12 Gy does not stimulate *Ifnβ1* production due to induction of Trex1-mediated inhibition 43. In MOC2 head and neck cancer cells, we observed a dose dependent increase in the expression of *Ifnβ1* and *Mx1* that peaked at day 1 following radiation (Figure 1A-B), which is consistent with prior reports 42, 43. Additionally, the magnitude of this effect at day 1 was sensitive to the dose of radiation, and we observed significantly greater increased expression of *Ifnβ1* and *Mx1* in cells treated with 12 Gy as compared to those treated with 2.5 Gy (p = 0.0007, 0.0025 respectively; Figure 1A-B). We observed similar trends with *Oas2* and *Oas3* in cells treated with 12 Gy with peak expression occurring at day 1 following radiation (Figure 1C-D). In contrast, we observed a delayed time course of expression of *Oas2* and *Oas3* in cells treated with 2.5 Gy with peak expression occurring 7 to 14 days following radiation (Figure 1C-D).

To determine whether these observations were generalizable to other cancer models we measured the effect of 2.5 Gy or 12 Gy EBRT on expression of *Ifnβ1*, *Mx1*, *Oas2*, and *Oas3* in B78 melanoma cells *in vitro*. We again confirmed a dose-dependent increase in the expression of *Ifnβ1* (day 1 2.5 Gy vs 12 Gy p = 0.0054, day 7 2.5 Gy vs 12 Gy p = 0.0008; Figure 1E)*,* however peak expression was observed later in the time course at day 7 following radiation. *Mx1* demonstrated similar kinetics with peak expression occurring day 7 following radiation (Figure 1F). Similar to MOC2, we observed peak expression of *Oas2* and *Oas3* occurring at day 1 following radiation in cells treated with 12 Gy (Figure 1G-H). Additionally, we observed a delayed time course of expression of *Oas2* and *Oas3* in cells treated with 2.5 Gy with peak expression occurring 7 to 14 days following radiation (Figure 1G-H). IFN1 activation was confirmed on the protein level using pIRF3 as a marker for activation. We observed an increase in pIRF3 level at day 1 and 7 following radiation in MOC2 and B78 respectively (Figure S1A-B). We then evaluated the timing of IFN1 activation in B16 melanoma. In B16 cells we observed a STING-dependent increase in the expression of *Ifnβ1* (Figure 1I), *Mx1* (Figure 1J)*, Oas2* (Figure 1K), and *Oas3* (Figure 1L) with peak expression occurring at day 7 following radiation.

**Figure 2:**
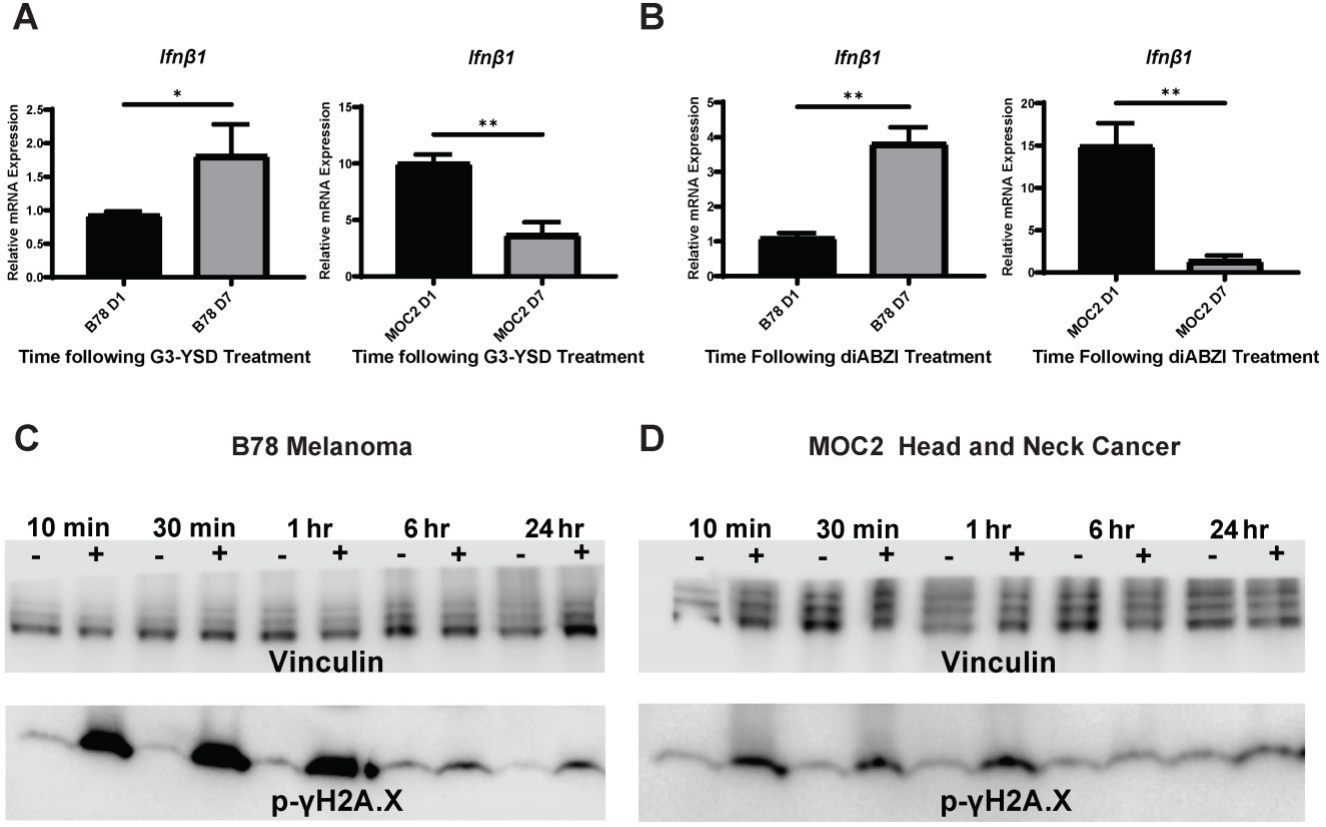
Time course differences between models of murine melanoma and head and neck cancer are mediated by kinetic differences in STING activation and not susceptibility to DNA damage. A,B) Cells were transfected with STING agonist G3-YSD and harvested either 1 or 7 days following transfection. qPCR was used to quantify expression of *Ifnβ* and is reported as fold change compared to untreated controls. N=3 per treatment group per timepoint. Student's t test was used to compare expression levels between time points. C,D) Cells were radiated with 12 Gy of EBRT *in vitro* and protein was isolated 10 min, 30 min, 1 hr, 6 hrs, or 24 hrs following radiation. Protein samples were probed for p-γH2A.X as a marker for DNA damage. Vinculin was used as a loading control. * indicates p-value < 0.05 and ** indicates p-value < 0.01.

We next sought to determine whether the difference in the time course of IFN1 activation in B78 and MOC2 cells were associated with corresponding differences in the susceptibility or response to DNA damage or in the kinetics of STING activation in these cell lines. B78 and MOC2 cells were exposed to single radiation doses of 0, 3, 6 or 9 Gy and their survival determined by colony formation assays. We observed no statistically significant difference between cell lines across all radiation doses tested, however the 9 Gy dose comparison trended towards significance (Figure S2). Using γH2A.X as a marker for DNA damage, we observed similar time courses in B78 and MOC2 cells in the emergence and subsequent repair of radiation-induced DNA damage with peak signal occurring 10-30 minutes following radiation and returning to baseline by 24 hours post radiation (Figure 2C-D). In contrast, when we transfected B78 and MOC2 cells *in vitro* with the STING agonist G3-YSD, we observed trends of IFN1 activation that mirrored the cell-line specific differences observed following radiation. Specifically, we observed peak expression of *Ifnβ1* occurring at day 7 following transfection with the G3-YSD STING agonist in B78 cells and at day 1 in MOC2 cells (Figure 2A). Treatment of B78 and MOC2 cells with the small molecule STING agonist diABZI resulted in similar findings (Figure 2B). These results suggest that the differences in time course of IFN1 activation between B78 and MOC2 reflect kinetic differences in the STING pathway between cell lines and not differences in DNA damage susceptibility or response.

### Changes in the expression of additional immune susceptibility markers following *in vitro* radiation exhibit variable dose and time dependence across tumor models

**Figure 3:**
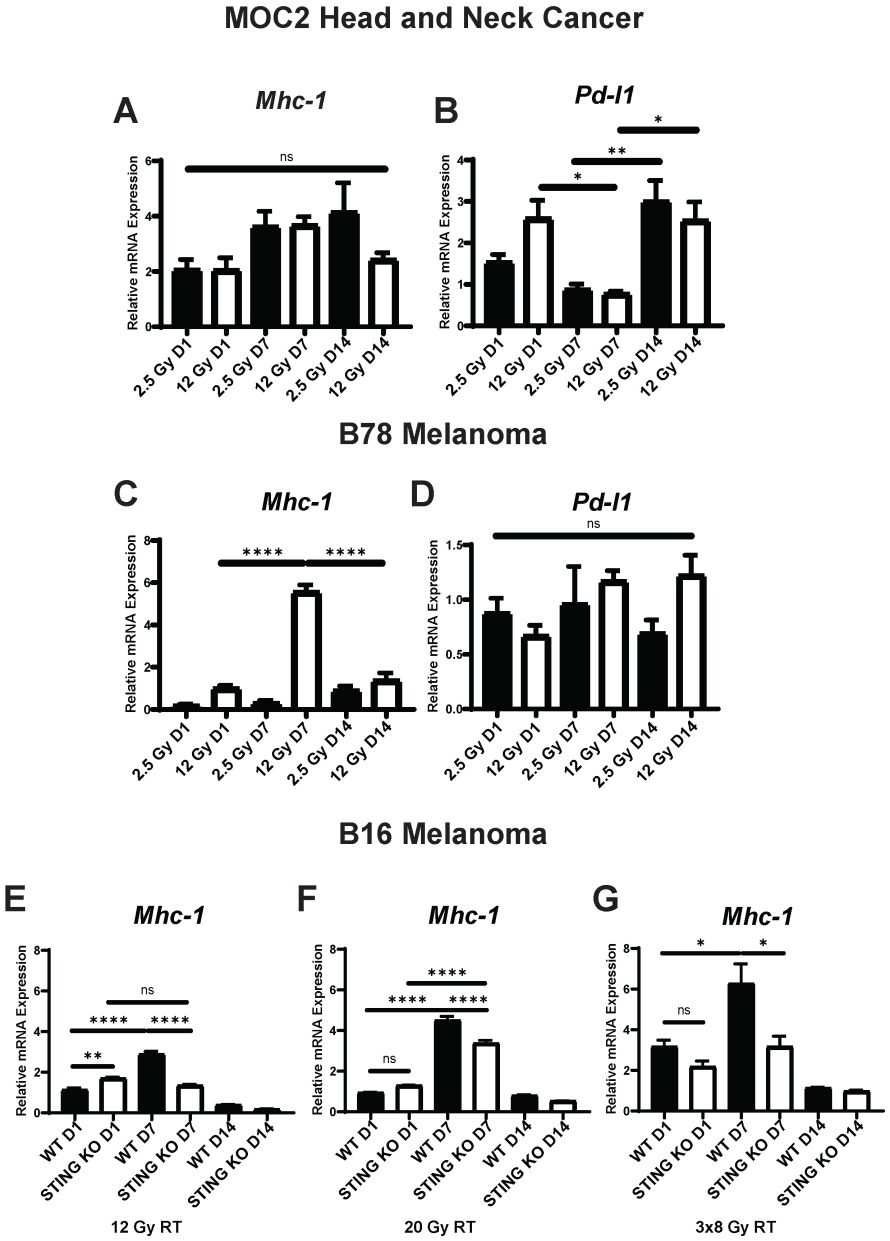
Expression of immune susceptibility markers exhibit variable dose and time dependence across tumor models following radiation *in vitro*. Cells were radiated with either 2.5 Gy or 12 Gy of EBRT and harvested 1, 7, or 14 days following radiation. qPCR was used to quantify gene expression and is reported as fold changed normalized to untreated controls. E-G) WT and STING KO B16 melanoma cells were radiated with either 12 Gy, 20 Gy, 3 daily fractions of 8 Gy and harvested either 1 or 7 days following the last fraction of radiation. N=5 per treatment group per timepoint. Two-way ANOVA with Tukey's HSD post hoc test was used to compare fold change in expression between groups. * indicates p-value < 0.05, ** indicates p-value < 0.01, *** indicates p-value < 0.001, and **** indicates p-value < 0.0001.

In addition to IFN1 activation, the expression of several other tumor cell markers of immune susceptibility are influenced by radiation, however, the dose responsiveness, time course, and STING-dependence of these effects has not been fully elucidated. For analysis, we selected the apoptotic death receptors *Fas* and *Dr5*, antigen presentation protein *Mhc-1*, and immune checkpoint ligand *Pd-l1* - each of which have been reported to be upregulated following radiation *in vivo* 18, 22, 44. In MOC2 cells radiated *in vitro* we observed a dose-dependent increase in the expression of *Fas* and *Pd-l1* that showed a biphasic time course with high expression at day 1 and again at day 14 following radiation (Figure S1A, Figure 3B). We observed similar expression levels of the death receptor *Dr5* across each dose and time point analyzed, with upregulation occurring at day 1 and sustained through day 14 following radiation (Figure S1B). Additionally, we observed similar expression levels of *Mhc1* between radiation doses with peak expression occurring at day 7 following radiation (Figure 3A,C).

As with the activation of IFN1, we observed distinct time courses for the activation of immune susceptibility markers in B78 cells, as compared to MOC2 cells. In B78 melanoma cells radiated *in vitro,* at all analyzed time points we observed comparable expression levels of the death receptor *Fas* with either 2.5 Gy or 12 Gy radiation and this expression was highest at day 14 after radiation for both dose levels (Figure S3C). However, the relative magnitude of change in expression compared to untreated controls was negligible with peak expression being 1.5-fold higher than untreated cells. We also observed comparable effects of 2.5 Gy and 12 Gy radiation on the expression of the death receptor *Dr5,* and this expression peaked at day 7 after radiation (Figure S3D). In contrast and in agreement with previous reports 17, 18, we observed a dose-dependent increase in the expression of *Mhc1* that peaked at day 7 following radiation (Figure 3C). Interestingly, we observed no change in expression of *Pd-l1* across dose levels or time points analyzed in B78 cells (Figure 3D), including additional analysis at day 2 and 3 following radiation (Figure S4A). *Pd-l1* protein expression was confirmed in B78 and MOC2 via western blot (Figure S1A-B). As in B78 melanoma, B16 melanoma displayed a delayed time course of *Mhc-1* expression (Figure 3E-G) with peak expression occurring at day 7 following radiation. Importantly, these observations in both B78 and MOC2 cells are in broad agreement with the published literature on the effects of radiation inducing the expression of immune susceptibility markers and yet simultaneously illustrate the potential for broad variation in the dynamic time course of these effects across tumor cell types.

IFN1 activation via the cGAS/STING pathway following radiation influences a diverse set of immune mechanisms including recruitment of immune cells and enhancement of dendritic cell antigen cross presentation and activation 43. However, the potential effects of the cGAS/STING pathway on radiation-induced changes in tumor cell expression of immune susceptibility markers has not been fully elucidated. We used a *Tmem173* -/- B16 cell line (STING KO), which lacks the *Tmem173* gene encoding STING, to evaluate the role of the cGAS/STING pathway and IFN1 activation in altering the expression of these immune susceptibility markers following radiation. We chose to focus on *Mhc1* as it demonstrated significant temporal and dose-dependent expression changes following radiation. We treated B16 WT and STING KO cells *in vitro* with 12 Gy EBRT. Additionally, we included a 20 Gy treatment as this dose has previously been shown to be optimal for induction of *Mhc1* expression 17. We also included a fractionated regimen of three fractions of 8 Gy, which has previously been suggested as an optimal regiment for IFN1 activation 43. Using qPCR to measure expression of *Mhc1*, we observed that even in the absence of STING, radiation induced a statistically significant increase in *Mhc1* expression at all 3 EBRT doses analyzed. The time course of this effect appeared identical to that in wild-type B16 cells, although the magnitude of this increase was significantly decreased in the B16 STING KO cells compared to wild type B16 at all 3 EBRT doses evaluated (Figure 3E-G). Interestingly, we observed no statistically significant increase in *Trex1* expression following 20 Gy of radiation (Figure S4B). Taken together, these results suggest that IFN1 influences peak expression of *Mhc-1* which may partially underlie the effect of IFN1 enhancement of antigen presentation 45, 46 and the distinct dose response relationships reported for IFN activation and induction of *Mhc-1* expression following radiation.

### *In vivo* IFN1 activation kinetics mirror *in vitro* IFN1 activation following radiation in murine melanoma and head and neck cancer (HNCC) models

**Figure 4:**
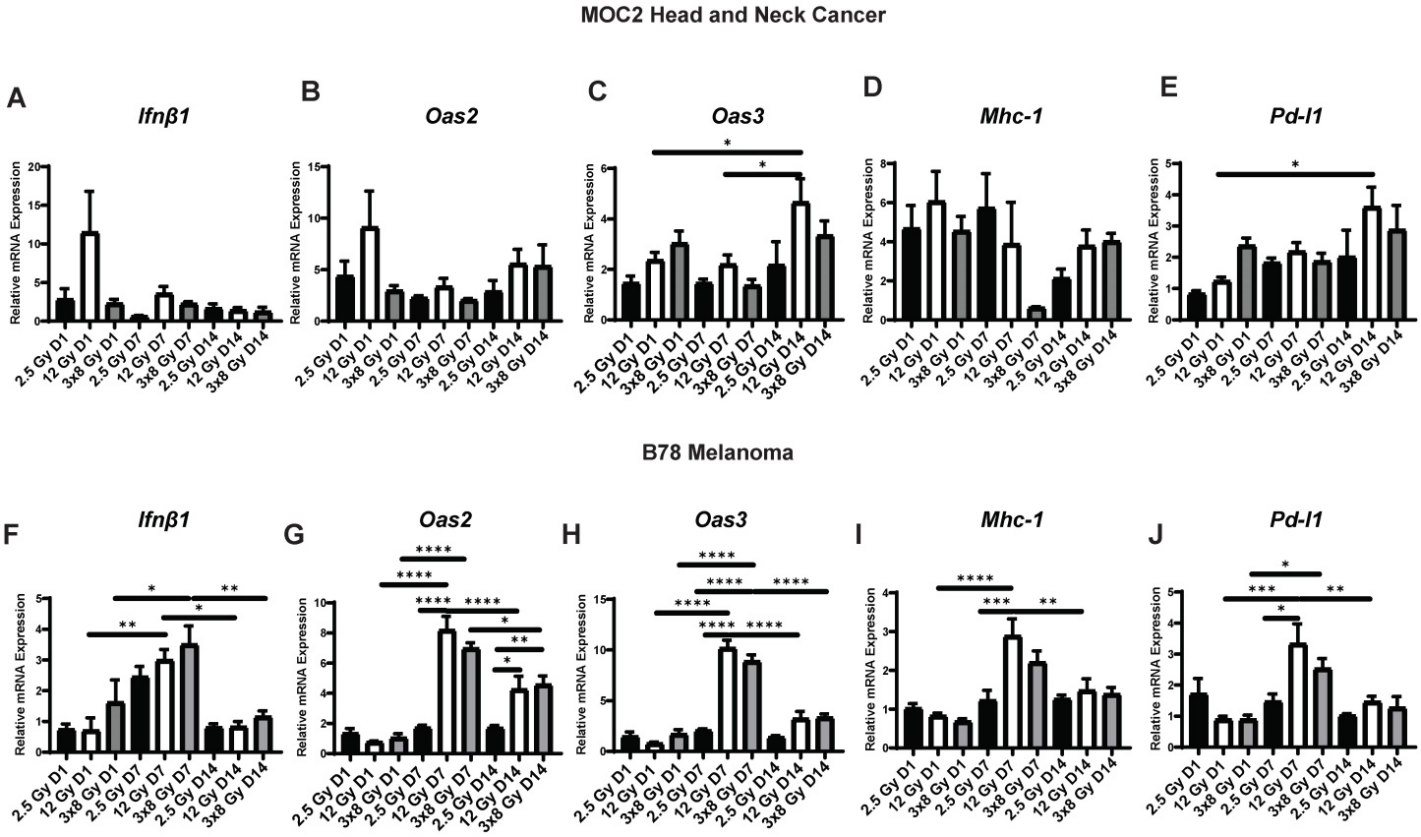
Radiation-induced IFN1 activation and immune susceptibility marker expression *in vivo* mirrors *in vitro* findings in murine melanoma and HNCC models. Tumors were grown to 100-150 mm^3^ and radiated with either 2.5 Gy, 12 Gy, or 3 daily fractions of 8 Gy and harvested 1, 7, or 14 days following the last fraction of radiation. qPCR was used to quantify gene expression and is reported as fold changed normalized to untreated controls. N=5 mice per treatment group per timepoint. Two-way ANOVA with Tukey's HSD post hoc test was used to compare fold change in expression between groups. * indicates p-value < 0.05, ** indicates p-value < 0.01, *** indicates p-value < 0.001, and **** indicates p-value < 0.0001.

Next, we sought to determine whether the dynamic changes in gene expression induced by radiation *in vitro* would also be observed *in vivo*. To do this, we engrafted C57BL/6 mice with syngeneic B78 murine melanoma or MOC2 head and neck cancer. When mean tumor volume reached 100-150 mm^3^ tumors were treated with a single fraction of 2.5 Gy or 12 Gy, 3 fractions of 8 Gy (once daily on three consecutive days), or sham radiation. We found that the time course of EBRT-induced changes in IFN1 activation and expression of immune susceptibility markers observed *in vivo* in both MOC2 head and neck cancer (Figure 4A-E) and B78 melanoma (Figure 4F-J) closely mirrored those observed *in vitro* after radiation. The only exception to this was *Pd-l1* expression in MOC2 which was elevated at day 1 but peaked later in the time course at day 14 *in vivo* compared to peaking at day 1 *in vitro*. Interestingly, there was no significant difference in IFN1 activation between 12 Gy and 3 × 8 Gy at any time point measured in either cell line.

### ^90^Y-NM600 activates IFN1 and induces expression of immune susceptibility markers with a magnitude and time course that is comparable to that observed following EBRT *in vivo*

**Figure 5:**
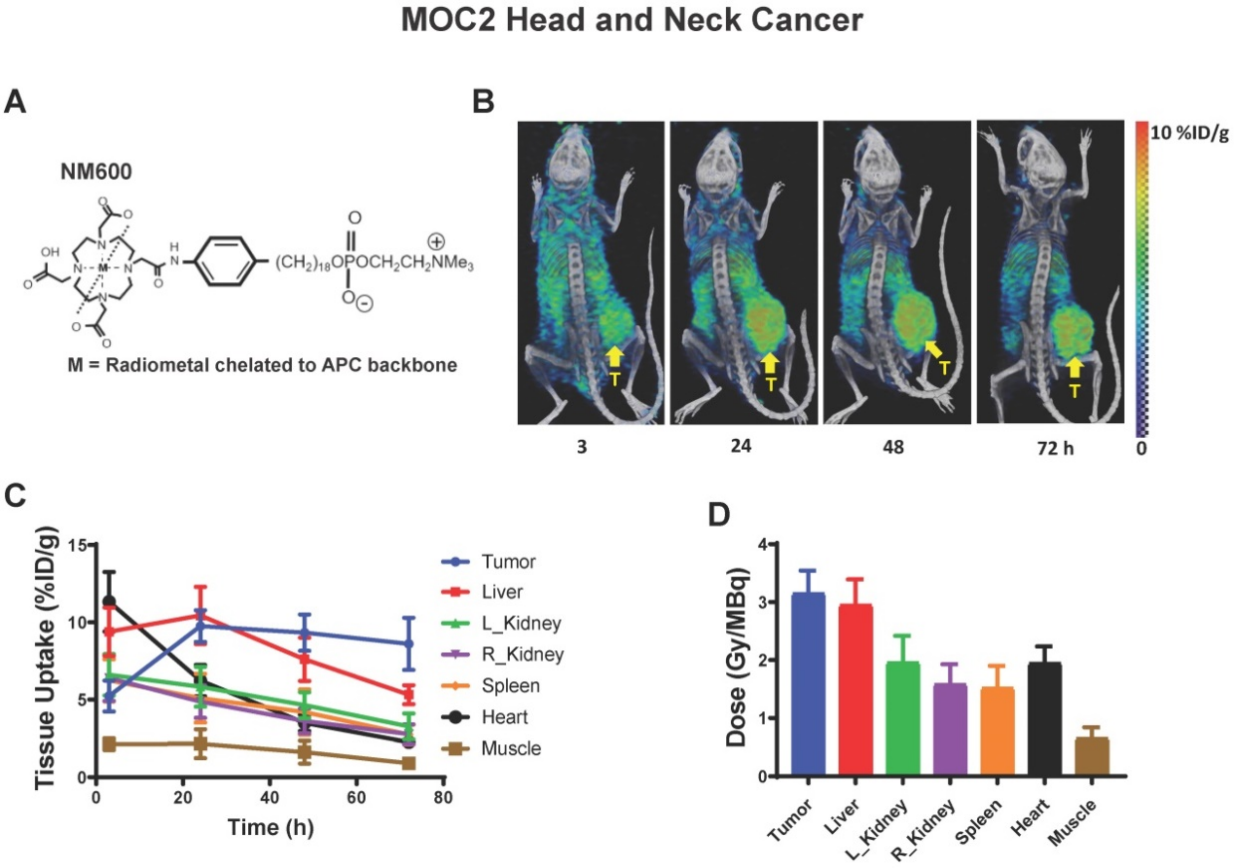
Dosimetry and biodistribution of ^86^Y-NM600 in MOC2 murine head and neck cancer. A) Schematic of NM600 B) PET/CT image time course of ^86^Y-NM600 3, 24, 48, and 72 hours following IV tail vein injection. Yellow arrow denotes tumor location. C) Biodistribution quantification comparing uptake of ^86^Y-NM600. D) Tissue specific dosimetry calculations 72 hours following IV tail vein injection.

With this emerging understanding of the dynamics of IFN1 activation and induced expression of immune susceptibility markers, we sought to understand whether these effects are activated by a TRT agent emitting radiation from a decaying radionuclide. Our group has pioneered the development of a novel class of TRTs using alkylphosphocholine (APCh) analogs (Figure 5A). Tumor cells contain an over-abundance of APChs 47 and we have shown that analogs to these display nearly universal and selective uptake and retention in melanoma, head and neck squamous cell carcinoma, and other murine and human tumors, regardless of anatomic location 23, 24, 37, 48, 49. We have previously demonstrated selective uptake and retention of NM600 in B78 melanoma tumors and reported on the biodistribution and dosimetry of ^90^Y-NM600 in this model 37.

**Figure 6:**
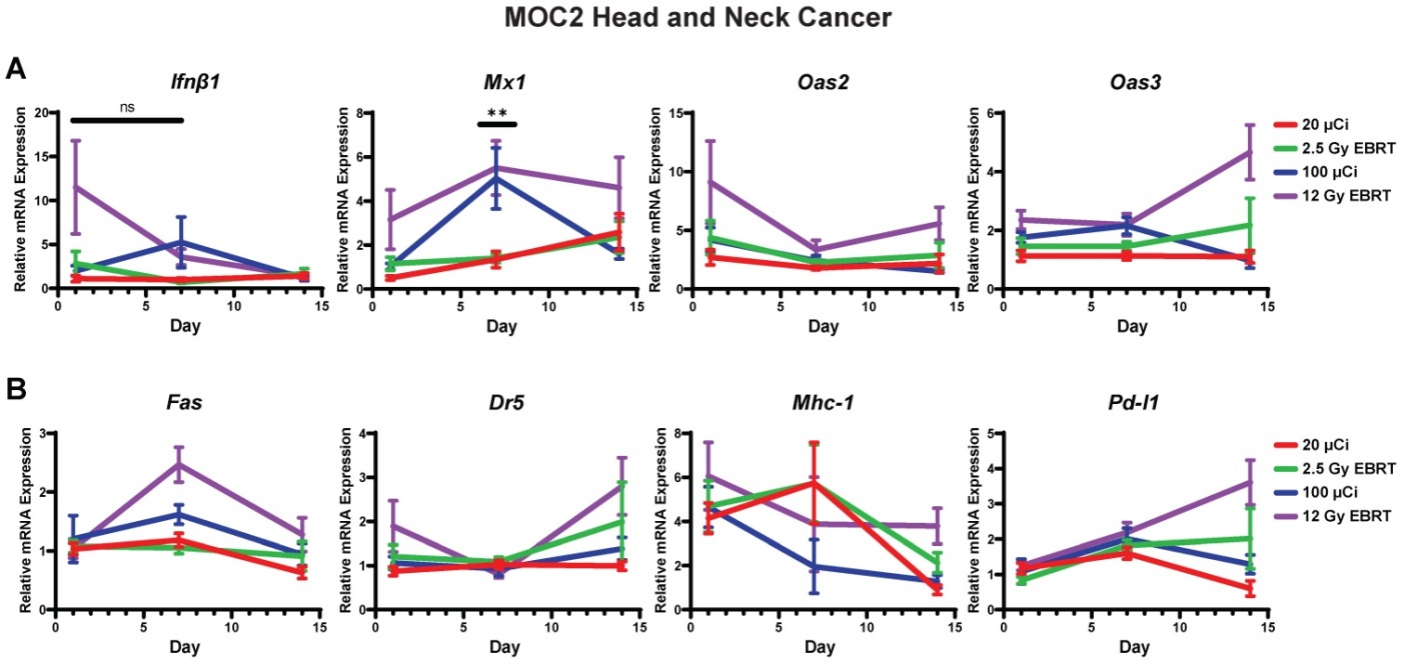
Time course of IFN1 activation in MOC2 head and neck cancer is delayed following ^90^Y-NM600 compared to EBRT *in vivo*. Tumors were grown to 100-150 mm^3^ and treated with either 20 µCi or 100 µCi of ^90^Y-NM600 (corresponding to ~ 2.5 Gy and 12 Gy tumor absorbed dose, respectively) and harvested 1, 7, or 14 days following injection. qPCR was used to quantify gene expression and is reported as fold changed normalized to untreated controls. EBRT expression data from Figure 3 is replotted here for reference. Two-way ANOVA with Tukey's HSD post hoc test was used to compare fold change in expression between ^90^Y-NM600 treated groups. ** mark where expression level differences generated by 2.5 Gy and 12 Gy of ^90^Y-NM600 are statistically significant, P < 0.01.

**Figure 7:**
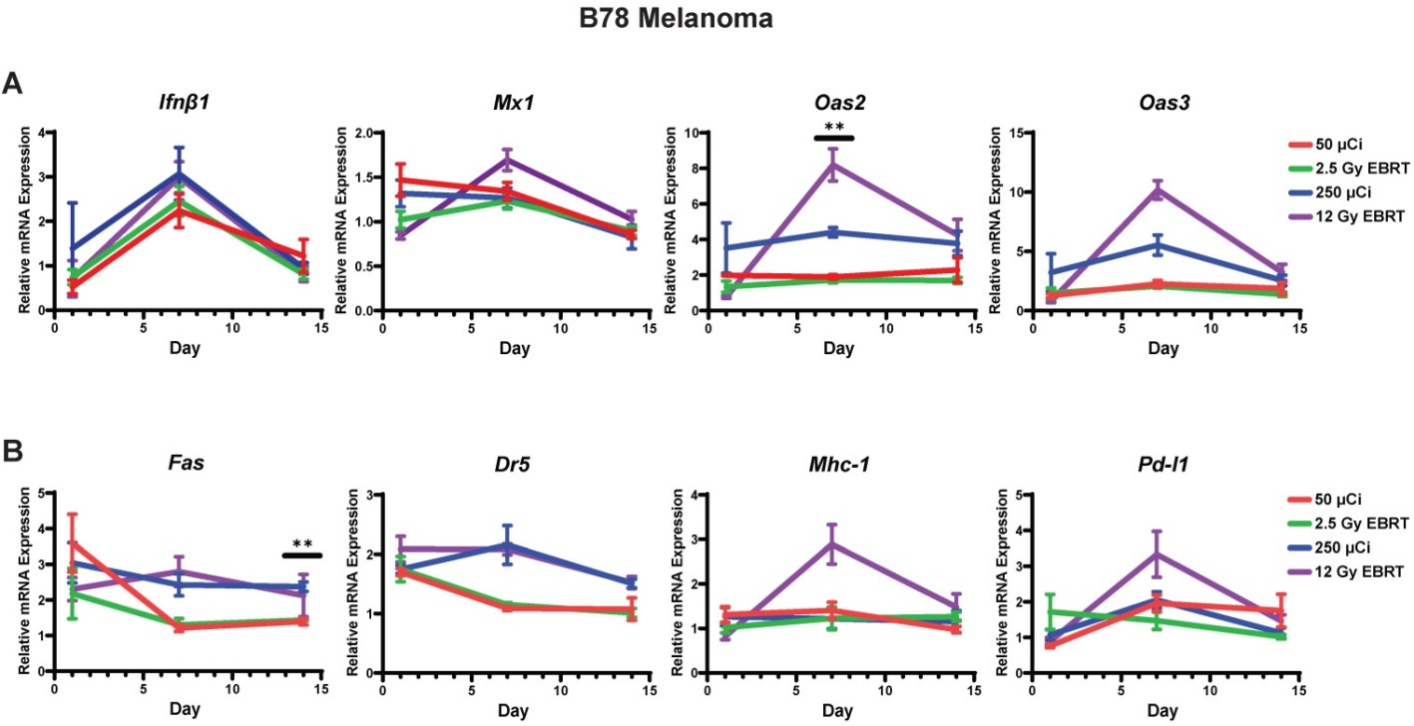
Time course of IFN1 activation in B78 melanoma is comparable following EBRT or ^90^Y-NM600 *in vivo*. Tumors were grown to 100-150 mm^3^ and treated with either 50 µCi or 250 µCi of ^90^Y-NM600 (corresponding to ~ 2.5 Gy and 12 Gy tumor absorbed dose, respectively) and harvested 1, 7, or 14 days following injection. qPCR was used to quantify gene expression and is reported as fold changed normalized to untreated controls. EBRT expression data from Figure 3 is replotted here for reference. Two-way ANOVA with Tukey's HSD post hoc test was used to compare fold change in expression between ^90^Y-NM600 treated groups. ** indicates expression level differences generated by 2.5 Gy and 12 Gy of ^90^Y-NM600 are statistically significant, P < 0.01.

Here, we confirmed selective uptake and retention of NM600 in the MOC2 tumor model (Figure 5B) and determined the biodistribution and dosimetry of ^90^Y-NM600 in this tumor model (Figure 5C-D). Using ^90^Y-NM600 to deliver 2.5 Gy or 12 Gy of radiation to MOC2 (20 µCi or 100 µCi injected activity) (Figure 6) or B78 (50 µCi or 250 µCi injected activity) (Figure 7) tumors *in vivo,* we observed that equal cumulative prescribed doses of radiation delivered from EBRT and ^90^Y-NM600 resulted in an equivalent magnitude of increased expression of *Ifnb1* and IFN1 stimulated genes *Mx1*, *Oas2*, and *Oas3* (Figure 6A; Figure 7A). We observed a comparable time course of gene expression changes between EBRT and ^90^Y-NM600 treated B78 melanoma tumors with peak expression occurring at day 7 following radiation. In radiated MOC2 tumors we observed a delayed time course following ^90^Y-NM600 compared to EBRT with peak expression occurring at day 7 and 1 respectively following radiation. This trend was similarly observed when comparing expression of the immune susceptibility markers *Fas*, *Dr5*, *Mhc-1*, and *Pd-l1* in these tumor models (Figure 6B, Figure 7B). These results suggest that ^90^Y-NM600 generates equivalent IFN1 activation and upregulation of immune susceptibility markers compared to an equivalent cumulative prescribed dose of EBRT, however, the time course of activation may be delayed, in the case of the MOC2 model, which may correspond to the delayed continuous delivery of radiation from the decaying TRT source.

### Splenocyte co-culture with tumor cells reveals STING-dependent synergy between ^90^Y-NM600 and anti-CTLA-4 blockade

**Figure 8:**
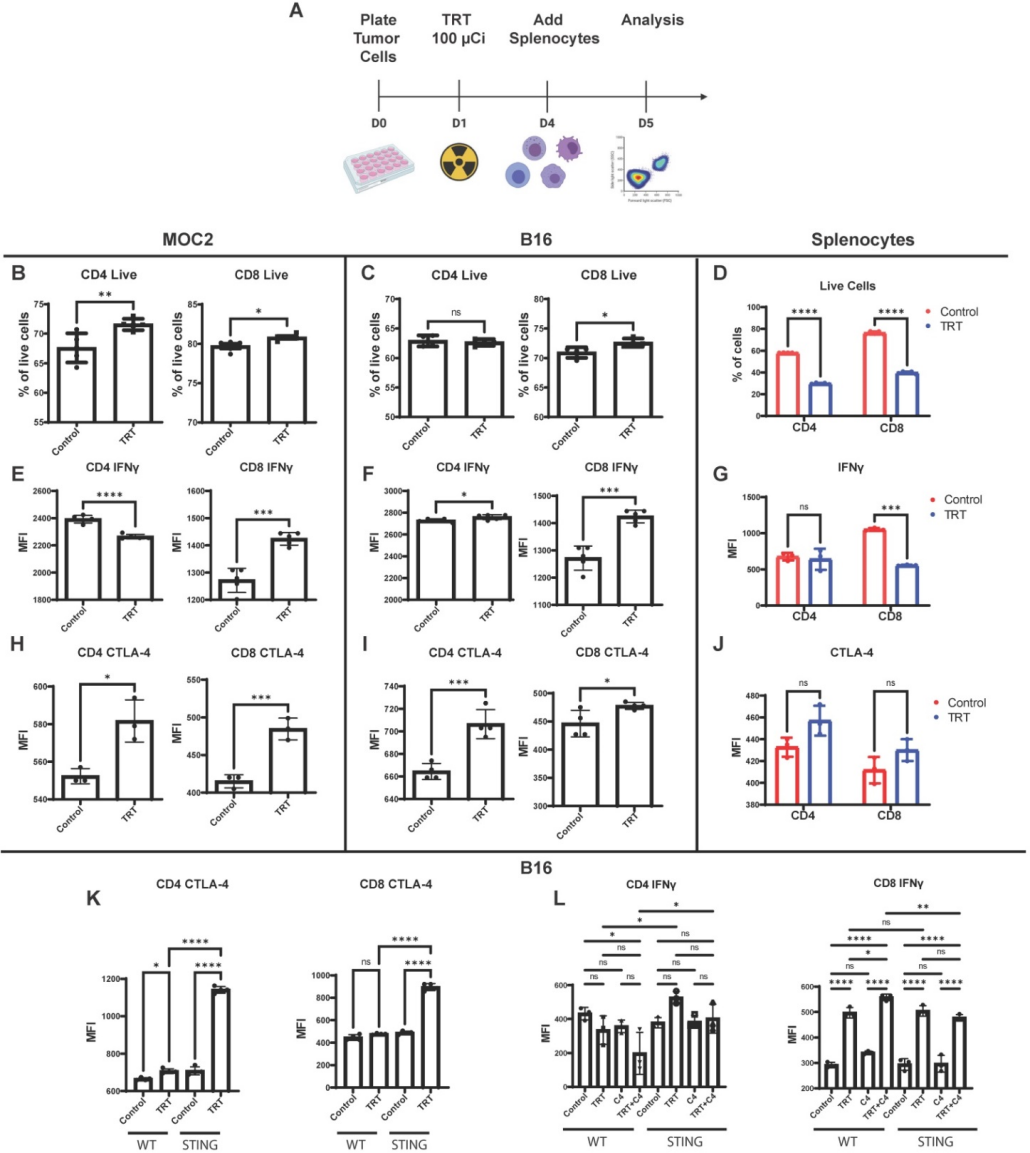
Co-culture with ^90^Y-NM600-treated tumor cells results in activation of IFNγ production in CD8+ T cells and increased expression of CTLA-4 (C4) on CD4^+^ and CD8^+^ T cells. (A) Tumor cells were plated on petri dishes and radiated with a prescribed cumulative dose of 12 Gy of ^90^Y-NM600 (140 µCi administered activity). Three days following addition of ^90^Y-NM600 to tumor cell culture media, splenocytes were added to the co-culture or empty culture plates without tumor cells, and 1 day following addition, splenocytes were harvested for analysis. For each culture condition CD4^+^ and CD8^+^ T cells were analyzed by flow cytometry for viability, activation status using IFNγ as a marker and expression of inhibitory CTLA-4 (schematic created with Biorender.com). (B-D) Quantification of number of CD4^+^ and CD8^+^ T cells alive at the end of co-culture with tumor cells (B, C) or without tumor cells (D). (E-G) Quantification of IFNγ expression on CD4^+^ and CD8^+^ T cells in co-culture with tumor cells (E, F) or without tumor cells (G). (H-J) Quantification of CTLA-4 expression on CD4^+^ and CD8^+^ cells in co-culture with tumor cells (H, I) or without tumor cells (J). (K) Tumor cell expression of STING suppresses the induction of CTLA-4 on CD4^+^ and CD8^+^ T cells upon co-culture of splenocytes with ^90^Y-NM600-treated B16 melanoma cells. (L) Addition of anti-CTLA-4 treatment increases expression of IFNγ on CD8^+^ T cells compared to ^90^Y-NM600 alone in a STING dependent fashion. Number of live cells and expression quantification was compared via Student's T test (A-J) or one-way ANOVA with Tukey's HSD post hoc test (K, L).

To further characterize the effects of ^90^Y-NM600 on tumor cells and to determine what effects these ^90^Y-NM600-induced changes have on immune cells, we co-cultured tumor cells with splenocytes in the presence of ^90^Y-NM600. *In vitro* dosimetry was determined using TLDs placed at the bottom of cell culture plates, and a serial dilution of known activities of ^90^Y-NM600 was added to generate a standard curve. B16 or MOC2 cells were treated with a prescribed cumulative dose of 12 Gy by culturing with ^90^Y-NM600 (140 µCi). Three days following addition of ^90^Y-NM600 to media (when tumor cells had received a little over 6 Gy), splenocytes were added. One day later splenocytes were harvested and analyzed by flow cytometry. In both B16 and MOC2 the number of live CD4 and CD8 cells was highest in the TRT group compared to control (Figure 8B-C). Conversely, splenocytes cultured without tumor cells in the presence of ^90^Y-NM600 showed a significant decrease in number of these populations (Figure 8D).

When co-cultured with either ^90^Y-NM600-treated MOC2 or ^90^Y-NM600-treated B16 cells, CD8+ T cells exhibited increased expression of IFNγ, as compared to those co-cultured with untreated control tumor cells (Figure 8E-F). This trend was not observed in splenocytes cultured without tumor cells (Figure 8G), suggesting ^90^Y-NM600-induced T cell activation requires signaling from radiated tumor cells. We investigated whether ^90^Y-NM600 also has an effect on inhibitory immune signals. When co-cultured with either ^90^Y-NM600-treated MOC2 or ^90^Y-NM600-treated B16 cells, both CD4+ T cells and CD8+ T cells demonstrated significantly increased expression of CTLA-4, as compared to those co-cultured with untreated control tumor cells (Figure 8H-I). A similar trend was observed with splenocytes cultured without tumor cells, but did not reach statistical significance (Figure 8J). We then compared ^90^Y-NM600 to EBRT and observed similar findings that the number of CD4 and CD8 cells alive at the end of the assay was higher in the ^90^Y-NM600 group compared to EBRT (Figure S5B-C). We observed no significant difference in expression of IFNγ in CD4 cells between EBRT and ^90^Y-NM600 (Figure S5D-E). However, EBRT significantly increased expression of IFNγ compared to ^90^Y-NM600 (Figure 5D-E). Additionally, we observed that ^90^Y-NM600 significantly increased expression of CTLA-4 on CD4 cells which may in part explain the difference in observed IFNγ expression between EBRT and ^90^Y-NM600 (Figure S5 F-G).

Using B16 WT and B16 STING KO cells, we sought to determine which of these effects of ^90^Y-NM600-treated B16 cells on T cells were dependent on tumor cell STING signaling. We observed a significant increase in CTLA-4 expression on CD4+ T cells and CD8+ T cells when co-cultured with ^90^Y-NM600-treated STING KO B16 cells, as compared to those co-cultured with ^90^Y-NM600-treated B16 WT cells (Figure 8K). On the other hand, we observed a similar degree of significantly increased IFN-gamma expression in CD8+ T cells after co-culture with either ^90^Y-NM600-treated B16 WT or ^90^Y-NM600-treated B16 STING KO cells (Figure 8L). This suggests that ^90^Y-NM600-treated tumor cells can activate CD8 cells through a STING-independent mechanism. Interestingly, in the co-culture of B16 cells and splenocytes, activation of IFN-gamma production among CD8+ T cells was increased by the addition of anti-CTLA-4 in the setting of ^90^Y-NM600-treated B16 WT cells, but not in the setting of ^90^Y-NM600-treated B16 STING KO cells (Figure 8L). This suggests that, while ^90^Y-NM600-treated tumor cells can activate IFN-gamma production independent of tumor cell expression of STING, the beneficial effects of anti-CTLA-4 in combination with ^90^Y-NM600 may be STING-dependent.

### ^90^Y-NM600 enhances anti-tumor response to dual immune checkpoint blockade in the MOC2 head and neck squamous cell carcinoma tumor model

We and others have previously shown that EBRT can enhance response to immune checkpoint inhibitors resulting in improved survival in animal models, and this effect is critically dependent on the activation of an IFN1 response via STING 1, 7, 8, 21, 22, 50. Given our observation that TRT can activate an IFN1 response equivalent to that achieved by equal dose EBRT, we hypothesized that TRT could augment response to dual immune checkpoint blockade with anti-CTLA-4 and anti-PD-L1. In an initial study we tested cumulative doses of 2.5 Gy (20 µCi) and 12 Gy (100 µCi) of ^90^Y-NM600 in combination with anti-CTLA-4 and found that a cumulative dose of 12 Gy of ^90^Y-NM600 was more effective in reducing tumor growth and extending survival compared to 2.5 Gy (Figure S6). Therefore, we used a cumulative dose of 12 Gy in subsequent studies. We chose to use a dual checkpoint blockade regimen given our observation that ^90^Y-NM600 treatment increased CTLA-4 expression on CD4+ T cells (Figure 8) and PD-L1 expression on tumor cells (Figure 3). To test this in a model of metastatic disease, we randomized mice bearing a “primary” right flank MOC2 tumor and a “secondary” left flank MOC2 tumor to treatment with PBS control, anti-CTLA-4 and anti-PD-L1 dual immune checkpoint blockade (ICI), 12 Gy tumor dose from ^90^Y-NM600 (100 µCi injected activity), 12 Gy tumor dose delivered to the primary tumor via EBRT, 12 Gy tumor dose from ^90^Y-NM600 together with ICI, or 12 Gy tumor dose delivered to the primary tumor via EBRT together with ICI. We monitored tumor growth and mouse survival. For additional analysis, we obtained pre-treatment and post-treatment (day 24 following treatment initiation) CT scans of mice from ^90^Y-NM600, ICI alone, and ^90^Y-NM600 + ICI treatment groups. We observed that the combination of ^90^Y-NM600 and ICI therapy resulted in a significant reduction in primary tumor growth (Figure 8B) compared to PBS control, ICI, and radiation monotherapies (^90^Y-NM600 + ICI vs ICI, p<0.001; ^90^Y-NM600 + ICI vs ^90^Y-NM600, p< 0.001; ^90^Y-NM600 + ICI vs EBRT, p< 0.001). The primary tumor growth reduction observed with ^90^Y-NM600 + ICI was comparable to EBRT + ICI (p= 0.171). Treatment with ^90^Y-NM600 + ICI resulted in a significant reduction in secondary tumor growth (Figure 8C) compared to all other treatment groups (^90^Y-NM600 + ICI vs EBRT + ICI, p= 0.002). These findings were further supported by CT scan analysis (Figure 8E-G). Additionally, combination ^90^Y-NM600 + ICI therapy resulted in a significant increase in median overall survival (Figure 8D) compared to monotherapies (^90^Y-NM600 + ICI vs ^90^Y-NM600, 29 days vs 19.5 days, p<0.001; ^90^Y-NM600 + ICI vs ICI, 29 days vs 17 days, p<0.001; ^90^Y-NM600 + ICI vs EBRT, 29 days vs 19.5 days, p<0.001), and EBRT + ICI (29 days vs 22 days, p<0.001).

## Discussion

We report the time course of IFN1 activation *in vitro* and *in vivo* following both EBRT and TRT in multiple tumor models. We observe that peak IFN1 activation can be delayed by a week following radiation and this time course may vary considerably across tumor models. Specifically, we detect peak IFN1 activation at day 1 following EBRT in the MOC2 head and neck squamous cell carcinoma model, and this contrasts with delayed peak activation at day 7 after EBRT in the B16 and B78 melanoma models. This activation of IFN1 is dependent on the STING pathway, and cell line-specific differences in the time course of IFN1 activation correlate with cell line-specific differences in the kinetics of cGAS/STING pathway activation and likely not susceptibility or response to DNA damage.

We further report on the time course of changes in the expression of select immune susceptibility markers (*Mhc1*, *Pdl1*, *Fas*, and *Dr5*) in tumor cells surviving radiation. Prior studies indicated that the expression of each of these markers is increased following radiation 18-20, 22. However, the time course of these changes and the effect of the cGAS/STING pathway on these changes had not been elucidated. Here, we confirm the increased expression of these markers following radiation but observe variation in the time course of this effect across tumor lines. We further demonstrate that the expression of STING influences the magnitude but is not necessary for the radiation-induced transcription of these immune susceptibility markers.

TRT is an expanding radiotherapy treatment modality with growing applications in clinical oncology. However, it remains largely unknown what effects TRT may have on the activation of IFN1 and the expression of immune susceptibility markers in tumor cells. We report a time-resolved analysis of IFN1 activation following ^90^Y-NM600 treatment at multiple dosing levels in two syngeneic murine models *in vivo*. We observed that the time course of IFN1 activation following ^90^Y-NM600 treatment was similar to that after equivalent cumulative prescribed dose EBRT in B78 melanoma and delayed in the MOC2 model compared to EBRT. This difference, which is observed in the MOC2 model, is likely the result of delayed continuous delivery of radiation from the decaying TRT source as well as the pharmacokinetics of ^90^Y-NM600 uptake in tumor during the first 24 hours following injection. This finding may not be applicable to the B78 model due to its inherent delayed STING pathway kinetics as seen with IFN1 activation occurring at day 7 following STING agonist treatment. This delay could mask any potential difference in IFN1 activation induced by ^90^Y-NM600 compared to EBRT and further studies will be required to confirm this finding. It is important to bear in mind, however, that these findings were collected using a single isotope and may be specific to ^90^Y, which has a relatively short 2.7 day half-life and emits β-particles with linear energy transfer of approximately 0.2 keV/µm, which is comparable to that of high energy photons 51. Future studies will further evaluate whether and how other clinically relevant radionuclides may activate an IFN1 response in tumor cells.

Robust preclinical data demonstrates that IFN1 production downstream of cGAS/STING activation following EBRT is critical for the synergy of EBRT with immune checkpoint blockade 1-3, 43. This preclinical data has inspired over 500 active clinical trials investigating radiation in combination with immune checkpoint blockade therapies 52. In these settings, EBRT is used to elicit an *in situ* vaccination effect, diversifying the adaptive anti-tumor T cell response 53, 54. The *in situ* vaccine effect of EBRT has been demonstrated in both preclinical and clinical settings 4, 5, 8, 10, 12, yet only in preclinical settings has this effect of EBRT been proven to augment response to anti-PD-1, anti-CTLA-4, or other immunotherapies. Clinical evidence supporting synergy between EBRT and immunotherapy is growing, and early findings are promising 10, 55, 56. However, this synergy has not yet been confirmed in multiple large, randomized trials. This may reflect differences between preclinical tumor models and the clinical circumstances in which EBRT *in situ* vaccination has been tested clinically. Specifically, most preclinical studies of radiation and immunotherapy have utilized murine models with either a single tumor or with a second tumor site that is recently engrafted and/or microscopic at the time of treatment 1, 7, 8, 21, 22, 50. Clinical studies demonstrate that tumor burden has a detrimental effect on the response to immune checkpoint blockade 57, 58, and such preclinical models therefore may poorly represent the clinical context of widespread metastatic disease in which EBRT and immunotherapies are commonly being tested clinically.

EBRT exerts effects on the tumor microenvironment through both STING-dependent and independent mechanisms. Our findings suggest that TRT exhibits effects on tumor cells as well as tumor-infiltrating immune cells that are both dependent on and independent of STING signaling. For instance, upregulation of IFNγ in CD8^+^ T cells in co-culture was independent of tumor cell STING signaling, occurring in both WT and STING KO cells. On the other hand, the ability of anti-CTLA-4 to synergize with TRT to further increase expression of IFNγ in CD8^+^ T cells was dependent on STING. Interestingly, we observed a significant increase in CTLA-4 expression in CD4^+^ T cells co-cultured with STING deficient tumor cells compared to WT, which may be due to alternative compensatory pathways. Further studies exploring the effects of TRT on tumor cell immune susceptibility and activation of IFN1 in both tumor cells and immune cells are warranted and may include exploration of alternative pathways to activate IFN1 59.

To capitalize on the immunogenic effects of radiation in patients with metastatic disease, it may be most effective to deliver radiation to all tumor sites. In this way, all tumor sites may be effectively immunomodulated by the breadth of mechanisms whereby radiation interacts with the tumor microenvironment and tumor cells to increase susceptibility to anti-tumor immunity 60. For patients with metastatic disease, delivering EBRT to all tumors, including radiographically occult sites, would require whole body EBRT. However, such large field radiation causes lymphopenia, which would render combination with immune checkpoint inhibitors futile 61, 62. As an alternative, using TRT to deliver radiation to all sites of disease may avoid immunosuppressive lymphopenia while inducing IFN1 activation at all tumor sites. Our data suggests that, at least in the case of ^90^Y-NM600, systemic administration of TRT activates an IFN1 response in tumor cells with a magnitude and time course that is comparable to that achieved by equivalent cumulative prescribed dose, locally administered EBRT. Importantly, as with EBRT, here we observe that this effect of TRT is correlated with increased response to immune checkpoint blockade resulting in improved survival with the combination of ^90^Y-NM600 and dual anti-CTLA-4 and anti-PD-L1 in the immunologically cold MOC2 syngeneic murine model of head and neck squamous cell carcinoma 32, 33. In future studies, it will be important to evaluate whether and how this favorable therapeutic interaction of TRT and immune checkpoint blockade may be optimized, particularly with respect to the variables of dose, radionuclide, and type of radioactive decay.

We acknowledge several weaknesses in our study. Notably, because of our desire to evaluate these effects in settings of intact host immunity, our data was obtained using syngeneic murine models. Murine models are mainstays of robust preclinical testing, and prior studies in murine and human cells show conserved effects of EBRT on the activation of IFN1 and increased expression of immune susceptibility markers. However, the time course of these effects in human cells will need to be explored further in future studies. In addition, the effects of TRT in mice may not accurately reflect those in humans or larger animal models due to the fixed range of radiation emitted by TRT sources and the distinct size difference in tumors and distance to organs at risk in mice and humans. Here, we report early data showing cooperative effects from combination of a TRT and immune checkpoint inhibition. This data offers a proof-of-concept that these treatment modalities may be effectively combined. Additionally, we acknowledge that with our treatment scheme and analysis the dose delivered by TRT at day 1 following injection is not equivalent to that delivered by EBRT. However, to be able to administer an equivalent dose of radiation with TRT at day 1 following injection requires specific activities that are beyond the limit of tolerability and could result in toxicities that introduce high potential for confounding.

We expect that the preclinical and clinical investigation of EBRT and/or TRT in combination with immunotherapies will continue to be a very active area of preclinical and clinical investigation. In order to maximize the translational potential of these studies to achieve clinical benefit, it will be critical to further develop a precise mechanistic understanding of the interaction between radiation and anti-tumor immunity. In the context of integrating therapeutic combinations of radiation and immunotherapy, it may be particularly critical to develop a time-resolved understanding of these mechanisms so that therapies can be appropriately dosed and sequenced. Building from our current data, further studies, particularly in metastatic settings, are urgently needed to enable a mechanism-guided approach to evaluating the potential therapeutic benefits of integrating EBRT and/or TRT with immune checkpoint blockade and/or other immunotherapies.

## Supplementary Material

Supplementary figures and tables.

## Figures and Tables

**Figure 9:**
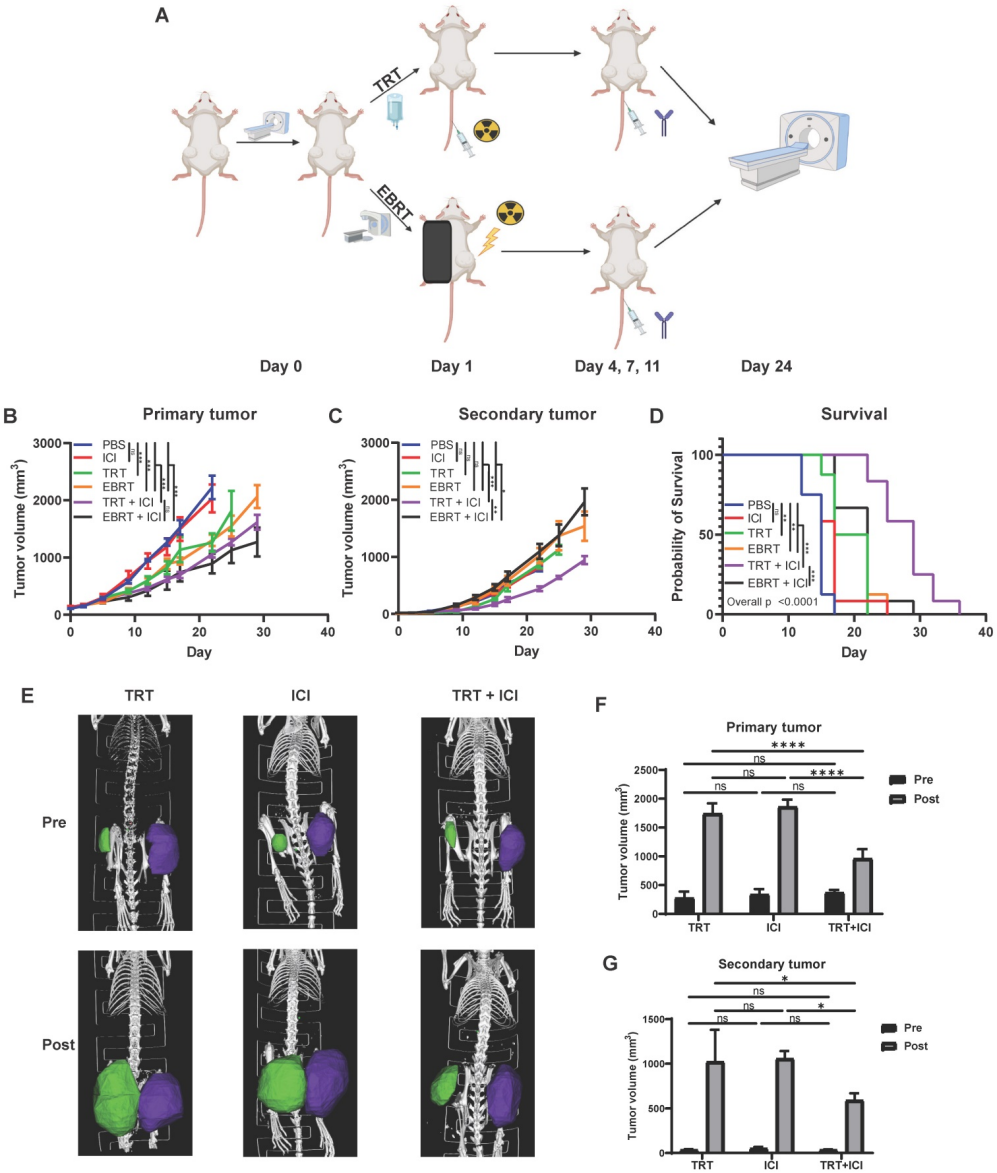
Combination of ^90^Y-NM600 and dual immune checkpoint inhibition reduces tumor growth and prolongs survival in mice bearing multiple MOC2 head and neck squamous cell carcinoma tumors. Mice were engrafted on the right flank with a “primary tumor” and one week later on the left flank with a “secondary tumor.” Mice bearing “primary” right flank and “secondary” left flank MOC2 tumors were randomized to PBS control, dual checkpoint blockade (ICI, anti-CTLA-4 + anti-PD-L1), 100 µCi of ^90^Y-NM600 (TRT, corresponding to a cumulative 12 Gy tumor prescription dose), 12 Gy of EBRT, combination TRT + ICI, or combination EBRT + ICI. Combination TRT + ICI therapy reduces primary tumor growth (B) compared to single agent therapy and is comparable to EBRT + ICI. Combination TRT + ICI therapy reduces secondary tumor growth (C) and significantly extends survival (D) compared to single agent therapy and EBRT + ICI. Prior to treatment (Pre) and at day 24 following treatment (Post), CT scans were obtained (E) and primary and secondary tumor volume was calculated using region of interest (ROI) analysis and were plotted for comparison (F, G). A linear mixed effect model was used to compare tumor volume over time. A log-rank test with Benjamini-Hochberg adjustment of p-values was used for pairwise comparison of overall survival and one-way ANOVA with Tukey's HSD post hoc test was used to compare tumor volumes calculated from CT scan ROI analysis, * indicates p-value < 0.05, ** indicates p-value < 0.01, and *** indicates p-value < 0.001. Schematic created with Biorender.com.

## Abbreviations

IFN1: type 1 interferon
STING: stimulator of interferon genes
cGAS: cyclic GMP-AMP synthase
EBRT: external beam radiotherapy
TRT: targeted radionuclide therapy
ICI: immune checkpoint inhibitor
HNCC: head and neck cancer cells
APCh: alkylphosphocholine
C4: anti-CTLA-4

## References

[1] Deng L, Liang H, Burnette B, Beckett M, Darga T, Weichselbaum RR (2014). Irradiation and anti-PD-L1 treatment synergistically promote antitumor immunity in mice. J Clin Invest.

[2] Deng L, Liang H, Xu M, Yang X, Burnette B, Arina A (2014). STING-Dependent Cytosolic DNA Sensing Promotes Radiation-Induced Type I Interferon-Dependent Antitumor Immunity in Immunogenic Tumors. Immunity.

[3] Weichselbaum RR, Liang H, Deng L, Fu YX (2017). Radiotherapy and immunotherapy: a beneficial liaison?. Nat Rev Clin Oncol.

[4] Twyman-Saint Victor C, Rech AJ, Maity A, Rengan R, Pauken KE, Stelekati E (2015). Radiation and dual checkpoint blockade activate non-redundant immune mechanisms in cancer. Nature.

[5] Demaria S, Ng B, Devitt ML, Babb JS, Kawashima N, Liebes L (2004). Ionizing radiation inhibition of distant untreated tumors (abscopal effect) is immune mediated. Int J Radiat Oncol Biol Phys.

[6] Demaria S, Bhardwaj N, McBride WH, Formenti SC (2005). Combining radiotherapy and immunotherapy: a revived partnership. Int J Radiat Oncol Biol Phys.

[7] Demaria S, Kawashima N, Yang AM, Devitt ML, Babb JS, Allison JP (2005). Immune-mediated inhibition of metastases after treatment with local radiation and CTLA-4 blockade in a mouse model of breast cancer. Clin Cancer Res.

[8] Dewan MZ, Galloway AE, Kawashima N, Dewyngaert JK, Babb JS, Formenti SC (2009). Fractionated but not single-dose radiotherapy induces an immune-mediated abscopal effect when combined with anti-CTLA-4 antibody. Clin Cancer Res.

[9] Formenti SC, Demaria S (2009). Systemic effects of local radiotherapy. Lancet Oncol.

[10] Formenti SC, Rudqvist NP, Golden E, Cooper B, Wennerberg E, Lhuillier C (2018). Radiotherapy induces responses of lung cancer to CTLA-4 blockade. Nat Med.

[11] Cai X, Chiu YH, Chen ZJ (2014). The cGAS-cGAMP-STING pathway of cytosolic DNA sensing and signaling. Mol Cell.

[12] Rudqvist NP, Pilones KA, Lhuillier C, Wennerberg E, Sidhom JW, Emerson RO (2018). Radiotherapy and CTLA-4 Blockade Shape the TCR Repertoire of Tumor-Infiltrating T Cells. Cancer Immunol Res.

[13] Sharabi AB, Nirschl CJ, Kochel CM, Nirschl TR, Francica BJ, Velarde E (2015). Stereotactic Radiation Therapy Augments Antigen-Specific PD-1-Mediated Antitumor Immune Responses via Cross-Presentation of Tumor Antigen. Cancer Immunol Res.

[14] Lin L, Kane N, Kobayashi N, Kono EA, Yamashiro JM, Nickols NG (2021). High-dose per Fraction Radiotherapy Induces Both Antitumor Immunity and Immunosuppressive Responses in Prostate Tumors. Clin Cancer Res.

[15] Dudzinski SO, Cameron BD, Wang J, Rathmell JC, Giorgio TD, Kirschner AN (2019). Combination immunotherapy and radiotherapy causes an abscopal treatment response in a mouse model of castration resistant prostate cancer. J Immunother Cancer.

[16] Wattenberg MM, Fahim A, Ahmed MM, Hodge JW (2014). Unlocking the combination: potentiation of radiation-induced antitumor responses with immunotherapy. Radiat Res.

[17] Reits EA, Hodge JW, Herberts CA, Groothuis TA, Chakraborty M, Wansley EK (2006). Radiation modulates the peptide repertoire, enhances MHC class I expression, and induces successful antitumor immunotherapy. J Exp Med.

[18] Werner LR, Kler JS, Gressett MM, Riegert M, Werner LK, Heinze CM (2017). Transcriptional-mediated effects of radiation on the expression of immune susceptibility markers in melanoma. Radiother Oncol.

[19] Reap EA, Roof K, Maynor K, Borrero M, Booker J, Cohen PL (1997). Radiation and stress-induced apoptosis: a role for Fas/Fas ligand interactions. Proc Natl Acad Sci U S A.

[20] Sheard MA, Vojtesek B, Janakova L, Kovarik J, Zaloudik J (1997). Up-regulation of Fas (CD95) in human p53wild-type cancer cells treated with ionizing radiation. Int J Cancer.

[21] Morris ZS, Guy EI, Francis DM, Gressett MM, Werner LR, Carmichael LL (2016). *In situ* Tumor Vaccination by Combining Local Radiation and Tumor-Specific Antibody or Immunocytokine Treatments. Cancer Res.

[22] Deng L, Liang H, Burnette B, Weicheslbaum RR, Fu YX (2014). Radiation and anti-PD-L1 antibody combinatorial therapy induces T cell-mediated depletion of myeloid-derived suppressor cells and tumor regression. Oncoimmunology.

[23] Morris ZS, Weichert JP, Saker J, Armstrong EA, Besemer A, Bednarz B (2015). Therapeutic combination of radiolabeled CLR1404 with external beam radiation in head and neck cancer model systems. Radiother Oncol.

[24] Weichert JP, Clark PA, Kandela IK, Vaccaro AM, Clarke W, Longino MA (2014). Alkylphosphocholine analogs for broad-spectrum cancer imaging and therapy. Sci Transl Med.

[25] Eke I, Ingargiola M, Förster C, Kunz-Schughart LA, Baumann M, Runge R (2014). Cytotoxic properties of radionuclide-conjugated Cetuximab without and in combination with external irradiation in head and neck cancer cells *in vitro*. Int J Radiat Biol.

[26] Nestor MV (2010). Targeted radionuclide therapy in head and neck cancer. Head Neck.

[27] Strosberg J, El-Haddad G, Wolin E, Hendifar A, Yao J, Chasen B (2017). Phase 3 Trial of (177)Lu-Dotatate for Midgut Neuroendocrine Tumors. N Engl J Med.

[28] Hofman MS, Violet J, Hicks RJ, Ferdinandus J, Thang SP, Akhurst T (2018). [(177)Lu]-PSMA-617 radionuclide treatment in patients with metastatic castration-resistant prostate cancer (LuPSMA trial): a single-centre, single-arm, phase 2 study. Lancet Oncol.

[29] Chen H, Zhao L, Fu K, Lin Q, Wen X, Jacobson O (2019). Integrin alphavbeta3-targeted radionuclide therapy combined with immune checkpoint blockade immunotherapy synergistically enhances anti-tumor efficacy. Theranostics.

[30] Choi J, Beaino W, Fecek RJ, Fabian KPL, Laymon CM, Kurland BF (2018). Combined VLA-4-Targeted Radionuclide Therapy and Immunotherapy in a Mouse Model of Melanoma. J Nucl Med.

[31] Czernin J, Current K, Mona CE, Nyiranshuti L, Hikmat F, Radu CG (2021). Immune-Checkpoint Blockade Enhances (225)Ac-PSMA617 Efficacy in a Mouse Model of Prostate Cancer. J Nucl Med.

[32] Judd NP, Allen CT, Winkler AE, Uppaluri R (2012). Comparative analysis of tumor-infiltrating lymphocytes in a syngeneic mouse model of oral cancer. Otolaryngol Head Neck Surg.

[33] Judd NP, Winkler AE, Murillo-Sauca O, Brotman JJ, Law JH, Lewis JS Jr (2012). ERK1/2 regulation of CD44 modulates oral cancer aggressiveness. Cancer Res.

[34] Haraguchi M, Yamashiro S, Yamamoto A, Furukawa K, Takamiya K, Lloyd KO (1994). Isolation of GD3 synthase gene by expression cloning of GM3 alpha-2,8-sialyltransferase cDNA using anti-GD2 monoclonal antibody. Proc Natl Acad Sci U S A.

[35] Bednarz B, Grudzinski J, Marsh I, Besemer A, Baiu D, Weichert J (2018). Murine-specific Internal Dosimetry for Preclinical Investigations of Imaging and Therapeutic Agents. Health Phys.

[36] Besemer AE, Yang YM, Grudzinski JJ, Hall LT, Bednarz BP (2018). Development and Validation of RAPID: A Patient-Specific Monte Carlo Three-Dimensional Internal Dosimetry Platform. Cancer Biother Radiopharm.

[37] Grudzinski JJ, Hernandez R, Marsh I, Patel RB, Aluicio-Sarduy E, Engle J (2019). Preclinical Characterization of. J Nucl Med.

[38] Marsh IR, Grudzinski J, Baiu DC, Besemer A, Hernandez R, Jeffery JJ (2019). Preclinical Pharmacokinetics and Dosimetry Studies of. J Nucl Med.

[39] Hernandez R, Walker KL, Grudzinski JJ, Aluicio-Sarduy E, Patel R, Zahm CD (2019). (90)Y-NM600 targeted radionuclide therapy induces immunologic memory in syngeneic models of T-cell Non-Hodgkin's Lymphoma. Commun Biol.

[40] Herzner AM, Hagmann CA, Goldeck M, Wolter S, Kubler K, Wittmann S (2015). Sequence-specific activation of the DNA sensor cGAS by Y-form DNA structures as found in primary HIV-1 cDNA. Nat Immunol.

[41] Ramanjulu JM, Pesiridis GS, Yang J, Concha N, Singhaus R, Zhang SY (2018). Design of amidobenzimidazole STING receptor agonists with systemic activity. Nature.

[42] Jin WJ, Erbe AK, Schwarz CN, Jaquish AA, Anderson BR, Sriramaneni RN (2020). Tumor-Specific Antibody, Cetuximab, Enhances the *In situ* Vaccine Effect of Radiation in Immunologically Cold Head and Neck Squamous Cell Carcinoma. Front Immunol.

[43] Vanpouille-Box C, Alard A, Aryankalayil MJ, Sarfraz Y, Diamond JM, Schneider RJ (2017). DNA exonuclease Trex1 regulates radiotherapy-induced tumour immunogenicity. Nat Commun.

[44] Vendetti FP, Karukonda P, Clump DA, Teo T, Lalonde R, Nugent K (2018). ATR kinase inhibitor AZD6738 potentiates CD8+ T cell-dependent antitumor activity following radiation. J Clin Invest.

[45] Zietara N, Łyszkiewicz M, Gekara N, Puchałka J, Dos Santos VA, Hunt CR (2009). Absence of IFN-beta impairs antigen presentation capacity of splenic dendritic cells via down-regulation of heat shock protein 70. J Immunol.

[46] Gessani S, Conti L, Del Cornò M, Belardelli F (2014). Type I interferons as regulators of human antigen presenting cell functions. Toxins (Basel).

[47] Snyder F, Wood R (1969). Alkyl and alk-1-enyl ethers of glycerol in lipids from normal and neoplastic human tissues. Cancer Res.

[48] Baiu DC, Marsh IR, Boruch AE, Shahi A, Bhattacharya S, Jeffery JJ (2018). Targeted Molecular Radiotherapy of Pediatric Solid Tumors Using a Radioiodinated Alkyl-Phospholipid Ether Analog. J Nucl Med.

[49] Grudzinski J, Marsh I, Titz B, Jeffery J, Longino M, Kozak K (2018). CLR 125 Auger Electrons for the Targeted Radiotherapy of Triple-Negative Breast Cancer. Cancer Biother Radiopharm.

[50] Morris ZS, Guy EI, Werner LR, Carlson PM, Heinze CM, Kler JS (2018). Tumor-Specific Inhibition of. Cancer Immunol Res.

[51] Behr TM, Béhé M, Stabin MG, Wehrmann E, Apostolidis C, Molinet R (1999). High-linear energy transfer (LET) alpha versus low-LET beta emitters in radioimmunotherapy of solid tumors: therapeutic efficacy and dose-limiting toxicity of 213Bi- versus 90Y-labeled CO17-1A Fab' fragments in a human colonic cancer model. Cancer Res.

[52] Jagodinsky JC, Harari PM, Morris ZS (2020). The Promise of Combining Radiation Therapy with Immunotherapy. Int J Radiat Oncol Biol Phys.

[53] Hammerich L, Binder A, Brody JD (2015). *In situ* vaccination: Cancer immunotherapy both personalized and off-the-shelf. Mol Oncol.

[54] Marabelle A, Tselikas L, de Baere T, Houot R (2017). Intratumoral immunotherapy: using the tumor as the remedy. Ann Oncol.

[55] Theelen W, Peulen HMU, Lalezari F, van der Noort V, de Vries JF, Aerts J (2019). Effect of Pembrolizumab After Stereotactic Body Radiotherapy vs Pembrolizumab Alone on Tumor Response in Patients With Advanced Non-Small Cell Lung Cancer: Results of the PEMBRO-RT Phase 2 Randomized Clinical Trial. JAMA Oncol.

[56] Fizazi K, Drake CG, Beer TM, Kwon ED, Scher HI, Gerritsen WR (2020). Final Analysis of the Ipilimumab Versus Placebo Following Radiotherapy Phase III Trial in Postdocetaxel Metastatic Castration-resistant Prostate Cancer Identifies an Excess of Long-term Survivors. Eur Urol.

[57] Topalian SL, Hodi FS, Brahmer JR, Gettinger SN, Smith DC, McDermott DF (2019). Five-Year Survival and Correlates Among Patients with Advanced Melanoma, Renal Cell Carcinoma, or Non-Small Cell Lung Cancer Treated with Nivolumab. JAMA Oncol.

[58] Sridharan V, Rahman RM, Huang RY, Chau NG, Lorch JH, Uppaluri R (2018). Radiologic predictors of immune checkpoint inhibitor response in advanced head and neck squamous cell carcinoma. Oral Oncol.

[59] Hou Y, Liang H, Rao E, Zheng W, Huang X, Deng L (2018). Non-canonical NF-kappaB Antagonizes STING Sensor-Mediated DNA Sensing in Radiotherapy. Immunity.

[60] Jagodinsky JC, Morris ZS (2020). Priming and Propagating Anti-tumor Immunity: Focal Hypofractionated Radiation for *in situ* Vaccination and Systemic Targeted Radionuclide Theranostics for Immunomodulation of Tumor Microenvironments. Semin Radiat Oncol.

[61] Diehl A, Yarchoan M, Hopkins A, Jaffee E, Grossman SA (2017). Relationships between lymphocyte counts and treatment-related toxicities and clinical responses in patients with solid tumors treated with PD-1 checkpoint inhibitors. Oncotarget.

[62] Ellsworth SG (2018). Field size effects on the risk and severity of treatment-induced lymphopenia in patients undergoing radiation therapy for solid tumors. Adv Radiat Oncol.

