# Supplementary material for: Temporal analysis of type 1 interferon activation in tumor cells following external beam radiotherapy or targeted radionuclide therapy
Source: Theranostics. 2021 Apr 15;11(13):6120–37. doi: 10.7150/thno.54881 (PMC8120207; doi:10.7150/thno.54881)
Supplement: Supplementary file 1 — Supplementary figures and tables. [file thnov11p6120s1.pdf]

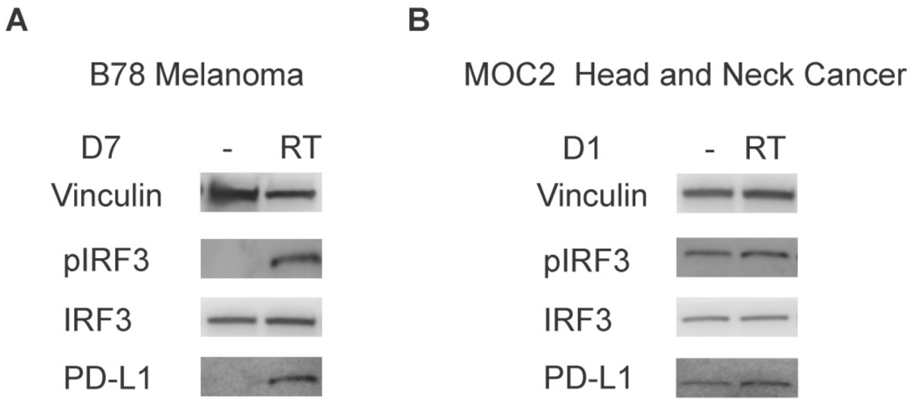

Figure S1. Radiation induces phosphorylation of IRF3 and increased expression of Pd-l1 protein *in vitro*. Cells growing in monolayer were irradiated with 12 Gy of EBRT and harvested at either day 1 or 7 following radiation, corresponding to the observed peak in expression of Ifnβ1 and Pd-l1 in B78 (day 7) and MOC2 (day 1). Protein samples were probed for pIFR3 as a marker for IFN1 activation or Pd-l1. Vinculin was used as a loading control.

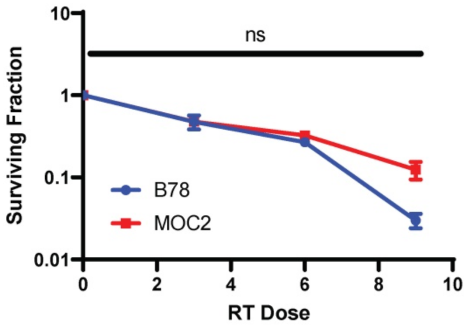

Dose	B78	MOC2	P-value	Significance
0 Gy	1	1	>0.9999	NS
3 Gy	0.475	0.478	>0.9999	NS
6 Gy	0.269	0.324	0.5981	NS
9 Gy	0.03	0.124	0.0793	NS

Figure S2. Radiosensitivity of B78 and MOC2 are comparable. Known numbers B78 and MOC2 cells in monolayer culture were irradiated with either 0 Gy, 3 Gy, 6 Gy or 9 Gy. After irradiation, cells were harvested and replated for clonogenic survival analysis. The log surviving fraction of control and irradiated colonies were calculated and plotted. One-way ANOVA with Tukey's HSD post hoc test was used to compare surviving fractions across dose levels.

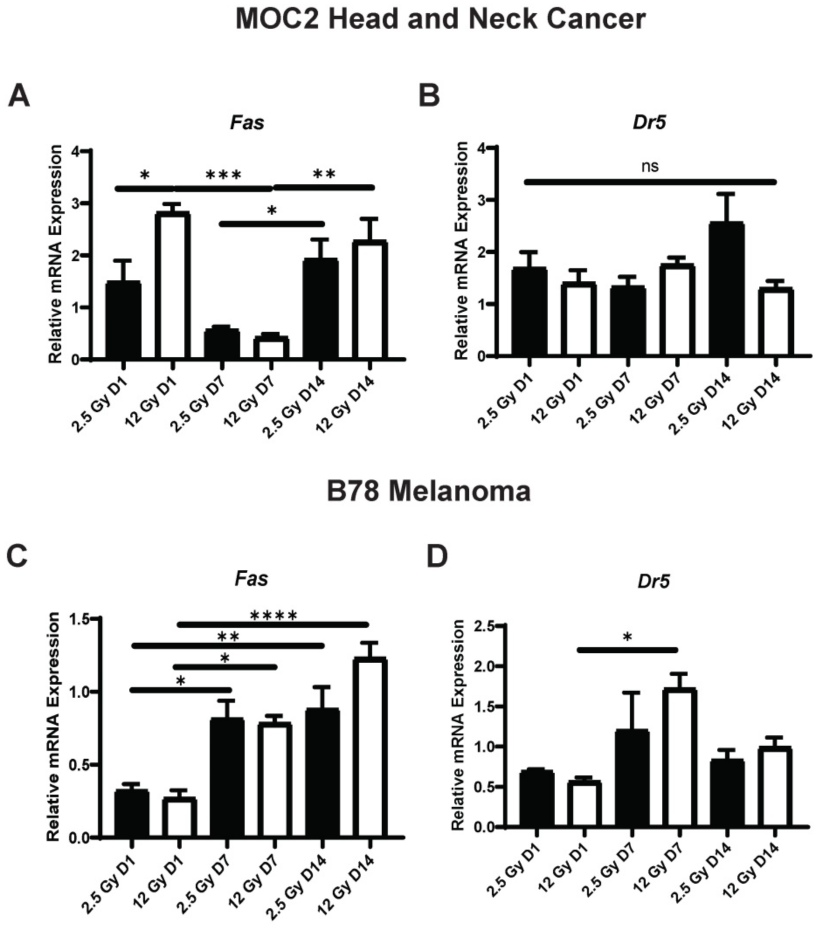

Figure S3. Time course of death receptor gene expression in murine models of melanoma and head and neck cancer following EBRT *in vitro*. Cells were radiated with either 2.5 Gy or 12 Gy of EBRT and harvested 1, 7, or 14 days following radiation. qPCR was used to quantify gene expression and is reported as fold changed normalized to untreated controls. N=5 per treatment group per timepoint. Two-way ANOVA with Tukey's HSD post hoc test was used to compare fold change in expression between groups.

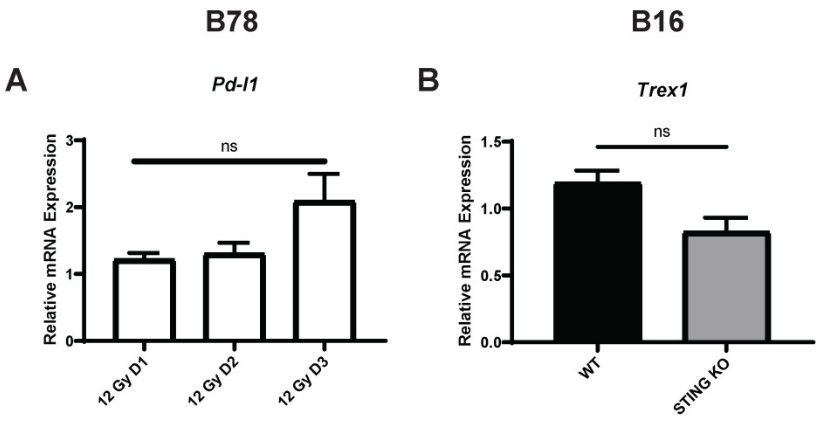

Figure S4. Time course of *Pd-l1* gene expression and analysis of *Trex1* induction in murine models of melanoma following EBRT *in vitro*. In the case of *Pd-l1* analysis (A) cells were radiated with 12 Gy and harvested 1, 2, or 3 days following radiation. For *Trex1* analysis (B) B16 WT and STING KO cells were radiated with 20 Gy and haversted 1 day later. qPCR was used to quantify gene expression and is reported as fold changed normalized to untreated controls. n=5 per treatment group per timepoint. One-way ANOVA with Tukey's HSD post hoc test was used to compare fold change in *Pd-l1* expression between groups and Student’s T test was used to compare *Trex1* expression between WT and STING KO.

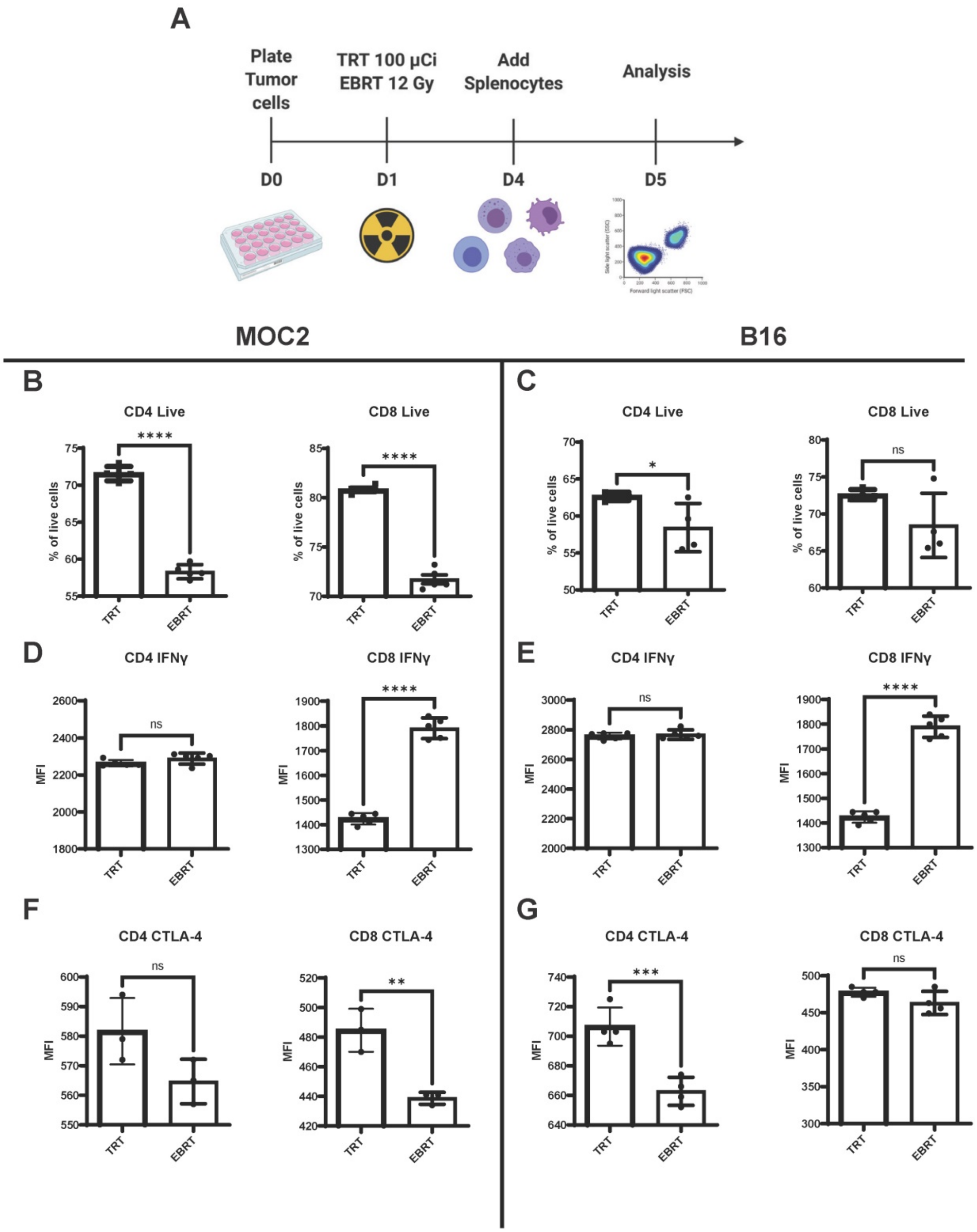

Figure S5. Tumor cells were radiated with a cumulative absorbed dose of 12 Gy of ^{90}Y-NM600 (140 µCi administered activity) or EBRT. Three days following radiation splenocytes were added to the co-culture or empty culture plates without tumor cells, and 1 day following addition, splenocytes were harvested for analysis. In each culture condition CD4^{+} and CD8^{+} T cells were analyzed for viability (B-D), activation status using IFNγ as a marker for activation (E-G), and expression of immune inhibitory CTLA-4 expression (H-J). Number of live cells and expression quantification was compared via Student's T test. Schematic created with Biorender.com

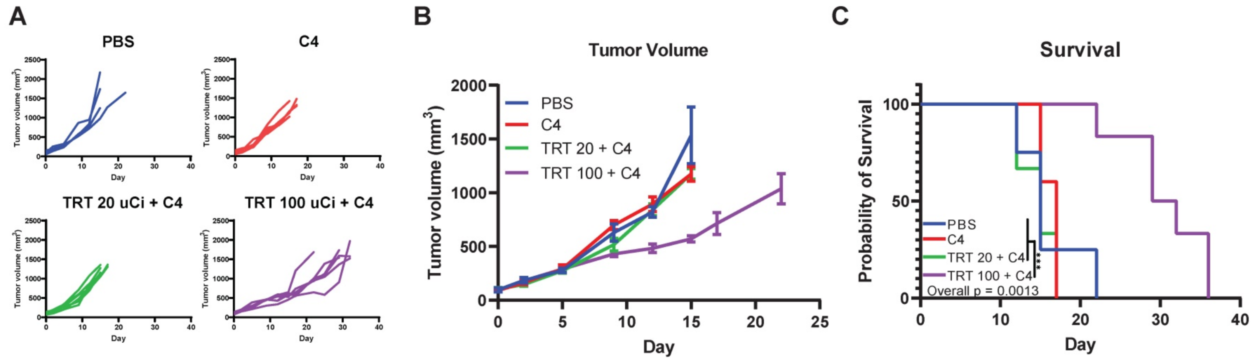

Figure S6. ^{90}Y-NM600 and anti-CTLA-4 combination therapy reduces tumor growth and prolongs survival in MOC2 head and neck cancer. MOC2 tumor bearing mice were randomized to PBS control, anti-CTLA-4 (C4), combination 20 µCi of ^{90}Y-NM600 and C4 (TRT 20 + C4, corresponding to ~ 2.5 Gy tumor absorbed dose), or combination 100 µCi of ^{90}Y-NM600 and C4 (TRT 100 + C4, corresponding to ~ 12 Gy tumor absorbed dose). Combination TRT 100 + C4 reduces tumor growth (A, B) and prolongs survival (C) compared to other treatment groups. A linear mixed model was used to compare tumor volume over time. A log-rank test with Benjamini-Hochberg adjustment of p-values was used for pairwise comparison of overall survival, * indicates p-value < 0.05, ** indicates p-value < 0.01, and *** indicates p-value < 0.001.

Table S1. Primer sequences

Gene	Forward Primer	Reverse Primer
Ifnβ1	TCCACCAGCAGACAGTGTTTC	TCAAGTGGAGAGCAGTTGAGG
Mx1	AGCTCACCTCCCACATCTGTAA	GCTTGCACTCTGATGACTGCTAT
Oas2	TAAGAGGCTGCTCCGATGGT	GACGTCAAGGTATGCATCTTGGT
Oas3	TTTCTCAGTCAAAGGCGTCCA	TCTATCCAGTGTTCTCCGTCTG
Fas	TACCGGAAAAGAAAGTGCTGGA	TGGTTTCACGACTGGAGGTT
Dr5	CCCATATAATGTGCAGGATGGC	TCGCTAGAATCTGGGACAGGA
Pd-l1	ATGTCAGGCCGAGGGTTATC	TCTCTTCCCACTCACGGGTT
Mhc-1	GTACCATCGCACCTGTCGG	CCGCGGACGCTGGATATAAA
Trex1	CCATTTCTCAGGGACTTCCA	AGCTCAGCTTTGCTCAGACC
Hprt	AGCCTAAGATGAGCGCAAGT	GGCCCACAGGACTAGAACACC
Pgk1	GGCATTCTGCACGCTTCAAA	CGACATTTTGGCAACACCGT
Tbp	GTTGGGCTTCCCAGCTAAGT	CACAAGGCCTTCCAGCCTTA
